# Indicators of mental disorders in UK Biobank—A comparison of approaches

**DOI:** 10.1002/mpr.1796

**Published:** 2019-08-08

**Authors:** Katrina A.S. Davis, Breda Cullen, Mark Adams, Anamaria Brailean, Gerome Breen, Jonathan R.I. Coleman, Alexandru Dregan, Héléna A. Gaspar, Christopher Hübel, William Lee, Andrew M. McIntosh, John Nolan, Robert Pearsall, Matthew Hotopf

**Affiliations:** ^1^ Institute of Psychiatry Psychology and Neuroscience King's College London London UK; ^2^ NIHR Biomedical Research Centre South London and Maudsley NHS Foundation Trust London UK; ^3^ Mental Health and Wellbeing, The Academic Centre, Gartnavel Royal Hospital University of Glasgow Glasgow UK; ^4^ Division of Psychiatry University of Edinburgh Edinburgh UK; ^5^ Peninsula Schools of Medicine and Dentistry Plymouth University Plymouth UK; ^6^ Devon Partnership NHS Trust Psychological Medicine, Exeter, UKUK Biobank, Office of the UKB Chief Scientist Edinburgh UK; ^7^ Office of the UKB Chief Scientist UK Biobank Edinburgh UK

**Keywords:** cohort study, diagnosis, epidemiology, mental disorder, online survey, UK Biobank

## Abstract

**Objectives:**

For many research cohorts, it is not practical to provide a “gold‐standard” mental health diagnosis. It is therefore important for mental health research that potential alternative measures for ascertaining mental disorder status are understood.

**Methods:**

Data from UK Biobank in those participants who had completed the online Mental Health Questionnaire (*n* = 157,363) were used to compare the classification of mental disorder by four methods: symptom‐based outcome (self‐complete based on diagnostic interviews), self‐reported diagnosis, hospital data linkage, and self‐report medication.

**Results:**

Participants self‐reporting any psychiatric diagnosis had elevated risk of any symptom‐based outcome. Cohen's *κ* between self‐reported diagnosis and symptom‐based outcome was 0.46 for depression, 0.28 for bipolar affective disorder, and 0.24 for anxiety. There were small numbers of participants uniquely identified by hospital data linkage and medication.

**Conclusion:**

Our results confirm that ascertainment of mental disorder diagnosis in large cohorts such as UK Biobank is complex. There may not be one method of classification that is right for all circumstances, but an informed and transparent use of outcome measure(s) to suit each research question will maximise the potential of UK Biobank and other resources for mental health research.

## 1 INTRODUCTION

Mental health is a major, and growing, contributor to disability worldwide (Whiteford, Ferrari, Degenhardt, Feigin, & Vos, 2015), prompting the need to take advantage of all available resources in order to progress the understanding of mental disorders and the interplay between mental and physical health (Prince et al., 2007). To this end, it is necessary to describe mental disorders and related traits in large‐scale epidemiological studies. The use of self‐report diagnosis, administrative data, and online surveys are potential sources of data on mental disorders that may be of use in this context, and so it is important to understand the advantages and limitations of these measures.

### 1.1 Considerations regarding indicators of mental health

Mental disorder diagnosis is a complex specialist task, requiring elucidation of symptoms, time course, and context (Casey & Kelly, 2007). It has not yet been possible to categorise mental disorders using pathology or aetiology, so, in order that there can be a common language, they have been systematised into syndromes based on signs and symptoms (Clark, Cuthbert, Lewis‐Fernández, Narrow, & Reed, 2017). However, it is not clear to what extent these syndromes reflect true disease entities, leaving difficulties at the boundaries both from normal variation and between different disorders (Kendell & Jablensky, 2003). Mental health research traditionally relies on lengthy structured or semistructured interviews to provide a “gold standard” highly reproducible syndromic diagnosis (Haro et al., 2006; Rucker et al., 2011), but these are costly to administer, placing a limit on sample sizes.

Common sources of mental disorder status in studies with large sample sizes are symptom scales or checklists, self‐reported clinical diagnoses and medication, and registries. Self‐report can be captured in a traditional interview, or using novel forms, such as online questionnaires, which vastly decrease costs of acquiring information (Andersson, Ritterband, & Carlbring, 2008). Registry data no longer come only from databases set up specifically for research but can be derived from administrative data. Data linkage to these sources offers benefits of a wider range of reports without the direct costs of acquiring data but raises problems of interpreting and validating those reports (Stewart & Davis, 2016).

Clinician diagnoses derived from self‐reported or data linkage should reflect the outcome of a nuanced clinical assessment, but those people who have received a diagnosis are those who have accessed services, whereas a large proportion of people with a mental disorder are never formally identified as such (Goldberg & Huxley, 1980). Passage into health care will depend upon the type and severity of illness and patient factors; receiving a diagnosis and treatment depends additionally on clinician and service factors. Such factors are vulnerable to age and cohort effects. For example, antidepressant treatment for those in whom the survey found symptoms of a common mental disorder in the previous year was almost three times more likely in 2009 (15.9%) than it had been in 1993 (5.7%; Spiers et al., 2016).

A retrospective enquiry adds recall bias for both symptoms and diagnoses. One study estimated that ability to recall a period of sadness likely to represent depression fell from 90% if it occurred in the last year to 41% if it occurred 10 years ago (Patten et al., 2012). This problem is not confined to mental health, because self‐report of clinician diagnosis of physical disorders including heart failure and previous cancer can be unreliable, leading mostly to underascertainment (Nord, Mykletun, & Fosså, 2003; Okura, Urban, Mahoney, Jacobsen, & Rodeheffer, 2004). It may be that mental disorders are also under‐reported due to perceived stigma of the disorder (Nevin, 2009; Simon & VonKorff, 1995).

### 1.2 Comparison of approaches in one resource

UK Biobank (UKB) is a research cohort for which over 503,328 people aged 49–60 enrolled in 2007–2010. This involved questionnaires, biosamples, and consent for linkage of routinely collected health care data and to take part in further waves of data collection (Sudlow et al., 2015).

The Mental Health Outcome Consortium was formed to develop mental health phenotyping in UKB. Mental disorder in this context might be both an outcome and a risk factor for other health outcomes. The consortium has focussed on two aspects: validating administrative secondary care diagnostic codes (Davis, Coleman, et al., 2018; Davis, Sudlow, & Hotopf, 2016) and designing an online Mental Health Questionnaire (MHQ) to identify symptom‐based outcomes (Davis, Bashford, et al., 2018). Some of the outcomes in the MHQ are based on diagnostic interviews and are analogous to mental disorder diagnoses (e.g., depression and generalised anxiety disorder). Others assess other aspects of mental health such as psychotic experiences (PEs) and self‐harm. Results of the UKB MHQ are available for 157,366 participants, representing 31% of the original UKB sample (Davis, Coleman, et al., 2018).

UKB now provides multiple indicators that could be used as a means to identify mental disorders, none of which represents a “gold‐standard” diagnosis against which the others can be validated. This could lead to confusion and dilemmas as to which measure to use for research. Although there have been studies that compare individual measures against a conventional gold standard, there are few resources that help guide the choice of imperfect measures in large studies such as UKB. The aim of this study is to compare four indicators of mental health and disorder in UKB for multiple outcomes, in order to guide future research in UKB and the design of similar studies.

## 2 METHODS

UKB is a major open science resource (Sudlow et al., 2015). Extensive data are already available on the 503,328 volunteers in UKB (UK Biobank, 2018), who responded to invitations sent by mail to people aged 40–69 who lived near to 22 assessment centres in England, Scotland, and Wales. The composition has been documented, and it has been noted that the volunteers are not representative of the population as a whole (Davis, Coleman, et al., 2018; Fry et al., 2017), in particularly under‐representing people with lower socio‐economic status, people with chronic illness, and smokers. This means that the data cannot be used to estimate population prevalence.

The methods used to develop and implement the online MHQ, participation, and features of nonparticipants are described in Davis, Coleman, et al. (2018). All UKB participants with a valid email address were sent a link in 2016–2017 (*n* = 339,092), and 46% of those invited submitted valid responses. People who responded had an average age of 65 years, and 57% were female. The questionnaire is still open on the website for participants to complete. We report findings based on the dataset released in August 2017 (*n* = 157,363; 31% cohort).

The four main methods of classifying these participants' mental health are symptom‐based outcomes, self‐report of diagnosis, hospital data linkage, and self‐report of medication. Brief explanations are provided below, with the full wording, criteria, cut‐offs, and code lists available in Appendices S1–S4. Table 1 shows examples of each method for four outcome groups that will be examined in Section 3. Some of these groups will have more closely aligned concepts that will allow comparison across methods, others will not. For example, PEs are not a true “symptom,” and most people who have these experiences do not have a psychotic disorder. Therefore, self‐report diagnosis and hospital data linkage of psychotic disorder should be viewed as complementary concepts to PE, whereas the depression outcome group are more akin to different methods of ascertaining the same concept.

**Table 1:** Summary of definitions for four measures (columns) that may be used to identify mental health outcomes for four example outcome groups (rows)

Mental health outcome	Symptom‐based outcome (see also Appendix S2)	Self‐report diagnosis	Hospital data linkage 1997–2015 (see also Appendix S3)	Self‐report medication 2007–2010 (see also Appendix S4)
Depression outcomes	Positive for major depressive disorder ever in MHQ (CIDI‐SF lifetime). Prevalence 24%	Endorsed clinician diagnosis of “depression” in MHQ. Prevalence 21%	Diagnosis of ICD‐10 depressive disorder (F32–33) on inpatient record. Prevalence 2%	Reported use of an antidepressant (prevalence 5%), antipsychotic (prevalence 0.3%), or lithium (prevalence 0.1%) at baseline.
Anxiety outcomes	Positive for generalised anxiety disorder ever in MHQ (CIDI‐SF lifetime). Prevalence 7%	Endorsed clinician diagnosis of “anxiety, nerves or generalised anxiety disorder” in MHQ. Prevalence 14%	Diagnosis of ICD‐10 neurotic disorders (F4×) on inpatient record. Prevalence 1%	Reported use of an antidepressant at baseline. Prevalence 5%
Bipolar affective disorder (BPAD) outcomes	Positive for wider bipolar criteria ever in MHQ (reflecting DSM‐IV hypomania/mania criteria). Prevalence 2%	Endorsed clinician diagnosis of “mania, hypomania, bipolar or manic‐depression” in MHQ. Prevalence 1%	Diagnosis of ICD‐10 mania or BPAD (F30–31) on inpatient record. Prevalence 0.2%	Reported use of lithium (prevalence 0.1%) or an antipsychotic (prevalence 0.3%) at baseline.
Psychotic experience (PE) outcomes	Endorsed one or more of four PEs ever (adapted CIDI PE lifetime).^a^ Prevalence 5%	Endorsed clinician diagnosis of “schizophrenia” or “other psychotic illness” in MHQ. Prevalence 1%	Diagnosis of ICD‐10 schizophrenia spectrum (F2×) or affective psychosis (F30.2, F31.2, F31.5, F32.3, and F33.3) on inpatient record. Prevalence 0.1%	Reported use of antipsychotic at baseline. Prevalence 0.3%

*Note.* Prevalence refers to criteria positive in this sample of 157,363 UK Biobank volunteers who completed the MHQ.

Abbreviations: CIDI‐SF, *Composite International Diagnostic Interview Short Form*; ICD‐10, *International Classification of Diseases 10th Revision*; MHQ, Mental Health Questionnaire.

a PEs are not true “symptoms” but outcome that can be related to psychotic disorder.

### 2.1 Symptom‐based outcomes

Lifetime depression, anxiety, bipolar affective disorder (BPAD), and PEs make up the lifetime “symptom‐based outcomes.” Lifetime measures were felt to be important to generate controls (“never had”) for genetic studies. Depression was assessed using the major depressive disorder section of the *Composite International Diagnostic Interview Short Form* (CIDI‐SF), and anxiety was assessed using the generalised anxiety disorder section of the CIDI‐SF, modified to provide lifetime history (Kessler, Andrews, Mroczek, Ustun, & Wittchen, 1998; Levinson et al., 2017). They were chosen on the basis of the ability to map on to DSM criteria, validity demonstrated by Levinson et al., and to maximise compatibility with studies internationally that were looking at the genetics of depression and anxiety. The CIDI‐SF uses DSM‐IV criteria but is also likely to represent a DSM‐5 diagnosis as these criteria are largely unchanged (American Psychiatric Association, 2013). Further questions assessed probable lifetime history of DSM‐defined hypomania/mania; criteria met for at least 1 week were used as the symptom‐based outcome for the BPAD outcome in this study. Lifetime PEs, not in themselves a disorder, were assessed using adapted questions from the CIDI (McGrath et al., 2015).

### 2.2 Self‐report of diagnosis

Participants were asked about clinician diagnoses of any medical condition at the baseline UKB interview and were specifically asked about mental disorders in the MHQ. We only use the prompted recall from the MHQ for this analysis. The questionnaire asked participants: “Have you been diagnosed with one or more of the following mental health problems by a professional, even if you don't have it currently?” Choices included “depression,” “anxiety, nerves or generalised anxiety disorder,” “mania, hypomania, bipolar or manic‐depression,” “schizophrenia,” and “other psychotic illness.”

### 2.3 Hospital data linkage

UKB (UK Biobank, 2014) has obtained structured diagnostic information from hospital admissions data to form a virtual hospital registry, combining Hospital Episode Statistics (HES), Scottish Morbidity Record (SMR 1a and 1b), and Patient Episode Database for Wales (PEDW) into a single dataset. Dates and completeness of coverage vary: PEDW dates back to 1999, HES to 1997, and SMR to 1981. HES and PEDW have mental health admissions in the same set as general hospital admissions, but Scotland do not. At the time of extraction, the Scottish mental health admissions (SMR‐04) were not available in UKB. Therefore, participants registered for the UKB in the two Scottish centres were excluded from the comparisons that involve hospital data linkage, leaving 146,813 participants in England and Wales. HES and PEDW use World Health Organization (WHO; 1992) *International Classification of Diseases 10th Revision* to categorise diagnosis. Cases were defined as having ever received an *International Classification of Diseases 10th Revision* diagnosis code relating to depression, anxiety, BPAD, or psychosis (see Table 1 or Appendix S3) in main or any secondary diagnoses. Psychosis codes included depression and BPAD where psychotic symptoms were specified.

### 2.4 Self‐report of medication

At baseline (2007–2010), 6 to 10 years before completion of the MHQ, UKB participants were asked whether they were taking any regular medication, and a nurse interviewer took the names of medication taken. A pre‐existing code list of antidepressants, antipsychotics, and lithium preparations was used to extract these data (see Appendix S4).

### 2.5 Data and analysis

The study used the UKB data release application number 16577 (application by G. B.), including valid MHQ data to June 2017 and hospital admission data 1997–2015, extracted and analysed using R version 3.4.3 (R Core Team, 2017) and code written by J. C. and K. D. (Davis, Coleman, et al., 2018). Full data are available from UKB (http://www.ukbiobank.ac.uk/register-apply/).

Confidence intervals are given at 95%, using Wilson's method for proportions. Cohen's *κ* was calculated as a measure of agreement between different methods of ascertainment for the same or equivalent outcomes.

### 2.6 Ethical approval

UKB has ethical approval from the North West—Haydock Research Ethics Committee (11/NW/0382) with MHQ approved as an amendment.

## 3 RESULTS

### 3.1 Self‐reported diagnosis

Table 2 is a cross‐tabulation of overlap between (a) self‐reported lifetime diagnosis and (b) symptom‐based outcomes. Percentages refer to the proportion of those with a self‐reported diagnosis (row) who met criteria for the specified symptom‐based outcomes (column). Of those that reported any mental disorder, 60% also met criteria for any symptom‐based outcome, whereas only 15% of those who reported no mental disorder met any criteria. The self‐report status (any vs. none) agreed with the symptom‐based status in 78%, with a *κ* of 0.46. Nearly 90% of people reporting BPAD or psychotic disorder met criteria for one or more symptom‐based outcome. Regardless which disorder was self‐reported, lifetime depression was the most likely symptom‐based outcome.

**Table 2:** Symptom‐based outcomes (SBOs, columns) and self‐reported diagnoses (SRs, rows)

[BLANK]		Overall		SBO No. of SBO ∩ SR (SBO|SR %)					
		*n*	Prev. in sample (%)	Depression	Anxiety	Wide bipolar definition	PE^a^	Any SBO	No SBO
Overall	*n*	157,363	NA	37,434	11,111	2,396	7,803	44,598	112,765
	Prev. in sample (%)	NA	NA	24	7	2	5	28	72
SR	Depression	33,424	21	20,714 (62%)	7,173 (21%)	1,314 (4%)	3,239 (10%)	22,651 (68%)	10,773 (32%)
	Anxiety	22,036	14	11,632 (53%)	5,711 (26%)	813 (4%)	2,051 (9%)	13,365 (61%)	8,670 (39%)
	BPAD	837	1	599 (72%)	248 (30%)	391 (47%)	358 (43%)	737 (88%)	100 (12%)
	Psychosis	723	1	491 (68%)	247 (34%)	187 (26%)	458 (63%)	635 (88%)	88 (12%)
	Panic disorder	8,704	6	4,555 (52%)	2,424 (28%)	399 (5%)	1,024 (12%)	5,273 (61%)	3,431 (39%)
	Eating disorder	1,851	1	1,048 (57%)	495 (27%)	101 (5%)	279 (15%)	1,201 (65%)	650 (35%)
	Personality disorder	385	<1	270 (70%)	171 (44%)	63 (16%)	141 (37%)	324 (84%)	61 (16%)
	Any self‐report	48,230	31	25,495 (53%)	9,081 (19%)	1,721 (4%)	4,255 (9%)	28,739 (60%)	19,491 (40%)
	No self‐report	109,133	69	11,938 (11%)	2,030 (2%)	675 (1%)	3,548 (3%)	15,859 (15%)	93,274 (85%)

*Note.* Numbers define participants with both stated symptom‐based outcome and self‐report (SBO ∩ SR), and % is the proportion of participants with given self‐report also having given symptom‐based outcome (SBO|SR). For definitions of symptom‐based‐outcomes, please see Appendix S2.

Abbreviations: BPAD, bipolar affective disorder; NA, not applicable; PE, psychotic experience.

a PEs are not true “symptoms” but outcome that can be related to psychotic disorder.

Depression, anxiety, and BPAD self‐reported diagnoses and symptom‐based outcomes are compared in Table 3. Depression outcomes had a *κ* of 0.46, anxiety outcomes have a *κ* of 0.28, and BPAD outcomes have a *κ* of 0.24.

**Table 3:** The overlap of self‐report (A) and symptom‐based outcome (B) for selected diagnoses, showing the intersection (A ∩ B), proportion overlap (B|A and A|B), and agreement (*κ*)

Mental Health Outcome	No. of self‐report (A)	No. of symptom‐based outcome (B)	No. of self‐report and symptom‐based outcome (A ∩ B)	% Symptom‐based outcome given self‐report (B| A)	% Self‐report given symptom‐based outcome (A| B)	*κ*
Depression	33,424	37,434	20,714	62	55	0.46
Anxiety	22,036	11,111	5,711	26	51	0.28
BPAD	837	2,396	391	47	16	0.24

Abbreviation: BPAD, bipolar affective disorder.

### 3.2 Hospital data linkage

Table 4 shows the partial overlap between the symptom‐based outcomes, self‐reported diagnosis, and hospital data linkage. The combination of three sources identified depression in 48,794 participants, but the hospital data linkage only identified 3,034 (6%) of these, most of whom (1,937) were also identified by both of the other two methods. Hospital data linkage identified 5% of anxiety cases and 9% of BPAD cases identified by any means. Of those with hospital data‐linkage diagnosis of psychotic illness (213), the symptom‐based outcome of PE was present in 67% (143).

**Table 4:** Identification of five mental health outcomes using symptom‐based outcomes, self‐report diagnosis, and hospital data linkage, for participants from England and Wales (*n* = 146,813)

Mental Health Outcome	Any	Symptom criteria (a)		Self‐report (b)		Hospital data linkage (c)		Combinations			
		Total	Alone	Total	Alone	Total	Alone	a ∩ b	a ∩ c	b ∩ c	All three
Depression	48,794	35,140 (72%)	15,472 (32%)	31,381 (64%)	11,919 (24%)	3,034 (6%)	257 (1%)	19,462 (40%)	2,143 (4%)	2,571 (5%)	1,937 (4%)
Anxiety	35,136	16,806 (48%)	8,324 (24%)	26,124 (74%)	17,264 (49%)	1,770 (5%)	555 (2%)	8,349 (24%)	704 (2%)	571 (2%)	571 (2%)
BPAD	2,709	2,247 (83%)	1,875 (69%)	783 (29%)	337 (12%)	245 (9%)	37 (1%)	364 (13%)	194 (7%)	120 (4%)	112 (4%)
PE^a^	7,686	7,390 (96%)	6,920 (90%)	684 (9%)	226 (3%)	213 (3%)	46 (1%)	434 (6%)	143 (2%)	131 (2%)	107 (1%)

*Note.* See Table 1 and Appendices S1–S4 for definitions. Total = no. of participants positive on given measure for given outcome (% positive for measure/positive for outcome). Alone = no. of participants that were positive for given measure and not for other measures in given outcome (% positive for this measure alone/positive for outcome). Combinations: *x* **∩** *y* = participants positive for both given criteria, irrespective of whether positive for third.

Abbreviations: BPAD, bipolar affective disorder; PE, psychotic experience.

a PEs are not true “symptoms” but outcome that can be related to psychotic disorder. Self‐report and hospital data linkage, in contrast, represent psychotic disorders.

### 3.3 Self‐reported medication

The snapshot view of selected self‐report medication use provided at the baseline assessment is shown in Tables 5a, 5b, 5c. Antidepressants were being taken by 8,616 (5.9%) participants, whereas antipsychotics and lithium were prescribed to less than 500 people each. Eighty‐three per cent of all people taking antidepressants were identified as having a lifetime history of depression through one of the three methods. Only half of the participants taking antipsychotics reported PEs or had a diagnosis of psychosis (229/470, 49%), although a further 35% (163/470) had an indicator of affective disorder. Lithium was almost confined to those identified as having an affective disorder—79% BPAD, 20% depression without evidence of BPAD.

**Table 5a:** Self‐report of any antidepressant for participants with depression and anxiety outcomes

Outcome	Depression	Anxiety	Nil
Symptom‐based outcome	5,352/35,140 (15.2%)	2,355/10,415 (22.6%)	
Self‐report diagnosis	6,378/31,381 (20.3%)	4,427/26,124 (16.9%)	
Hospital data linkage	1,492/2,858 (52.2%)	533/1,770 (30.1%)	
Self‐report antidepressant given above criteria	7,137/47,278 (15.1%)	5,123/31,071 (16.5%)	923/88,706 (1.0%)
		Excluding depression556/10,829 (5.1%)	
Any criteria given self‐report antidepressant	7,137/8,616 (82.8%)	Excluding depression556/8616 (6.5%)	923/8616 (10.7%)

*Note.* See Table 1 and Appendices S1–S4 for definitions. Self‐reported psychotropic use at baseline against psychiatric indication by three criteria: symptom‐based outcome, self‐report diagnosis, and hospital data linkage. % = proportion of cases screening positive for each criteria who reported medication use, except bottom row. Bottom row shows proportion of all participants reporting medication use who screened positive for each disorder.

**Table 5b:** Self‐report of any antipsychotic for participants with PEs or psychotic disorder, BPAD, and depression outcomes

Outcome	PE^a^	BPAD	Depression	Nil
Symptom‐based outcome	203/7,390 (2.7%)	103/2,247 (4.6%)	300/35,140 (0.9%)	
Self‐report	163/684 (23.8%)	135/783 (17.2%)	277/31,381 (0.9%)	
Hospital data linkage	84/213 (39.4%)	68/245 (27.8%)	105/2,858 (3.7%)	
Self‐report antipsychotic given above criteria	229/7,686 (3.1%)	161/2,709 (5.9%) Excluding PE 42/1,890 (2.2%)	354/47,278 (0.7%) Excluding PE and BPAD 121/41,359 (0.3%)	78/95,879 (0.1%)
Any criteria given self‐report antipsychotic	229/470 (48.7%)	Excluding PE 42/470 (8.9%)	Excluding PE and BPAD 121/470 (25.7%)	78/470 (16.6%)

*Note.* See Table 1 and Appendices S1–S4 for definitions. Self‐reported psychotropic use at baseline against psychiatric indication by three criteria: symptom‐based outcome, self‐report diagnosis, and hospital data linkage. % = proportion of cases screening positive for each criteria who reported medication use, except bottom row. Bottom row shows proportion of all participants reporting medication use who screened positive for each disorder.

Abbreviations: BPAD, bipolar affective disorder; PE, psychotic experience.

a PEs are not true “symptoms” but outcome that can be related to psychotic disorder. Self‐report and hospital data linkage, in contrast, represent psychotic disorders.

**Table 5c:** Self‐report of lithium prescription for participants with BPAD and depression outcomes

Outcome	BPAD	Depression	Nil
Symptom‐based outcome	73/2,247 (3.2%)	127/35,140 (0.4%)	NA
Self‐report	119/783 (15.2%)	111/31,381 (0.4%)	NA
Hospital data linkage	67/245 (27.3%)	50/2,858 (1.7%)	NA
Self‐report lithium given above criteria	131/2,709 (4.8%)	146/47,278 (0.3%) Excluding BPAD 34/45,195 (0.1%)	1/98,909 (0.0%)
Any criteria given self‐report lithium	131/166 (78.9%)	Excluding BPAD 34/166 (20.5%)	1/166 (0.6%)

*Note.* See Table 1 and Appendices S1–S4 for definitions. Self‐reported psychotropic use at baseline against psychiatric indication by three criteria: symptom‐based outcome, self‐report diagnosis, and hospital data linkage. % = proportion of cases screening positive for each criteria who reported medication use, except bottom row. Bottom row shows proportion of all participants reporting medication use who screened positive for each disorder.

Abbreviations: BPAD, bipolar affective disorder; NA, not applicable.

### 3.4 Combinations

Table 4 shows the results of combining symptom‐based outcomes, self‐reported diagnosis, and hospital data linkage in an additive manner for depression, anxiety, and BPAD. In all disorders, symptom‐based outcomes, self‐report, and hospital data linkage each contribute unique cases—but in different proportions for each disorder.

Combinations of outcomes for the common mental disorders of depression and anxiety are further explored in Table S1 and accompanying text. The symptom‐based outcomes were positive for depression or anxiety in 37,629 participants. Self‐reported or data‐linkage diagnosis of depression or anxiety or self‐reported antidepressant medication is positive in 47,321 participants, including 25,920 (55%) who were positive and 21,401 (45%) who were negative on lifetime symptom‐based outcomes.

## 4 DISCUSSION

In this study, we have compared methods of ascertainment for mental health outcomes in UKB from the position that none is equivalent to the outcome of a gold‐standard psychiatric interview. This situation is common in large nonspecialist research resources, and there is a need for resources to help with decision making when researchers are faced with a choice of imperfect measures.

We found that the magnitude of the overlap between the measures differed depending on the disorders. Depression outcomes were the most prevalent and had the most overlap between self‐report and symptom‐based outcomes (*κ* = 0.46). The proportion of participants with symptom‐based outcome who self‐reported a diagnosis was 55%, similar to the 61% of people of a similar age in a German study who were positive for lifetime depression on the Structured Clinical Interview for the DSM Axis‐I (SCID‐I) who self‐reported a diagnosis (Stuart et al., 2014).

A self‐reported diagnosis of “anxiety, nerves or generalised anxiety disorder” had less overlap with the corresponding symptom‐based outcome (*κ* = 0.28), a symptom‐based outcome for depression (53%) being more likely than anxiety (26%). Combining depression and generalised anxiety may be an acceptable strategy in population studies, where the concepts are largely overlapping (Gask, Klinkman, Fortes, & Dowrick, 2008), and in our data, this led to an improvement in agreement between self‐report and symptom‐based outcomes over anxiety, but not depression (*κ* = 0.46).

The conventional models of BPAD, with dramatic and disabling symptoms, would predict a high proportion to have been formally identified, but our symptom‐based outcome of BPAD was deliberately fairly wide to facilitate research into the wider spectrum of BPAD (Phillips & Kupfer, 2013) and would include many people who would meet the DSM criteria for BPAD type II as well as BPAD type I. People with BPAD type II will be less likely to be formally diagnosed or require inpatient treatment and hence will be less commonly identified by a hospital data linkage. Of those with BPAD symptom‐based outcome, 16% self‐reported clinician diagnosis and 9% had data‐linkage diagnosis. Self‐report diagnosis is somewhat higher in this study than in a similar Finnish population study (Perälä et al., 2007) where only 6% of those positive for the CIDI‐BPAD outcome self‐reported a diagnosis. This may be evidence of a cohort effect of different diagnostic behaviour or patient awareness between countries or over time.

PE and psychotic disorder are not equivalent, but complementary categories. We found that PE was almost 10 times more common than psychotic disorder reported by the participant and/or hospital data linkage (prevalence of PE 4.7% vs. psychotic disorder diagnosis 0.5%). The Finnish study (Perälä et al., 2007) found the rates of PE and psychosis diagnosis to be 3.0% and 3.3%, respectively. The lower prevalence of PE may be partly due to the mode of administration being interview, as PEs are more likely to be endorsed in self‐completed measures (Linscott & Van Os, 2013). The higher levels of diagnosis of a psychosis diagnosis may be partly because the registry used in the Finnish study goes back further in time but may also be related to participation bias. The Finnish study was a modest size study aiming at representativeness, with a participation rate of 93% of those selected, whereas UKB followed a different model, requesting volunteers from the community (Davis, Coleman, et al., 2018; Fry et al., 2017): People with an enduring psychotic disorder may have been less willing and/or able to volunteer.

Of the three self‐reported medication classes investigated, antidepressants were the most commonly reported. Even so, antidepressant prescription could only identify 15–17% of people with those symptom‐based outcomes of depression and anxiety. This is inevitable given the snapshot nature of the ascertainment of medication, the “treatment gap” (Kohn, Saxena, Levav, & Saraceno, 2004), and appropriate management of lifetime mental disorder without medication. Surprisingly, only 49% of those taking antipsychotics were positive on a measure of PE or psychosis, 35% had an affective disorder, and 13% neither. This fits with literature on the extended and off‐label prescribing of antipsychotics (Carton et al., 2015; Pringsheim, Gardner, & Patten, 2015).

### 4.1 Method of ascertainment

Symptom‐based outcomes do not require participants to have accessed care to detect a disorder, making them potentially the most sensitive out of the measures we compared, although the retrospective nature is likely to reduce sensitivity for distant episodes. By analysing participant responses to particular questions, it may also be possible to also look at subtypes or specific phenotypes or manipulate thresholds. Symptoms were collected using CIDI‐SF modules. The CIDI was created for the WHO programme and supported by them, although the short form is not currently supported by the WHO. Such measures are popular in surveys (McDowell, 2006; van Ballegooijen, Riper, Cuijpers, van Oppen, & Smit, 2016), although they can be overinclusive as they lack the ability to rule out other causes of the same symptoms (e.g., thyroid disturbance mimicking anxiety). Alternatives to the CIDI‐SF may have different, possibly better, performance—but this has not been tested.

Administration of self‐report diagnostic scales online is now an established practice (Andersson et al., 2008; Nguyen, Klein, Meyer, Austin, & Abbott, 2015), but there are generally less validation data available for measures administered electronically or via the Internet (Buchanan, 2003; van Ballegooijen et al., 2016). The performance of the CIDI‐SF depression module that was administered in the online MHQ has however been positively validated in at least two independent studies (Carlbring et al., 2002; Levinson et al., 2017).

Self‐reported clinician diagnosis is an easily obtainable measure, which allowed the MHQ to ask about a wide range of outcomes. As predicted, the diagnosis prevalence was lower than the symptom‐based outcome prevalence in the MHQ in most categories. The exception was generalised anxiety—which may be related to the wording of the question regarding anxiety diagnosis being vague. The presence of self‐reported diagnosis was associated with a greater risk of all symptom‐based outcomes, not just for equivalent outcomes, which reflects the co‐morbidities between disorders often unrecognised (Oiesvold et al., 2013; Whiteford et al., 2015). Another sources of self‐reported diagnosis in UKB are those reported during the baseline assessment. On that occasion, participants were not prompted to recall specific diagnoses and had to disclose them face to face. The prevalence of self‐reported mental prevalence was lower on that occasion, with depression reported by only 6.5%, as opposed to 21% at the MHQ. This is likely to do with the prompted recall but may also be due to stigma during a face‐to‐face interview and new diagnoses since baseline.

The hospital data linkage provided by UKB leverages national statistics to identify outcomes that are commonly documented in hospital admissions. The nature and patient pathway of mental disorders mean only the most severe cases are likely to be the cause of an admission (Goldberg & Huxley, 1980). Moreover, these episodes may have happened many decades ago, before 1997 when the data for England start. Most mentions of mental disorder will therefore be secondary diagnoses in participants admitted to hospital with other problems, which have not been specifically validated (Davis, Coleman, et al., 2018). In this study, the low numbers identified in hospital data linkage, with high levels of lifetime symptom‐based outcomes in those individuals, suggest a specific but insensitive measure. Registries based on data linkage to outpatient attendance or primary care consultations may give a more sensitive measure, although it is likely to be more complex to define cases given the myriad of coding types in these records (John et al., 2016; Spiranovic, Matthews, Scanlan, & Kirkby, 2016).

The use of self‐reported medication data is potentially problematic. Bias in recall of medication is very common, perhaps more so in psychotropics (Gnjidic, Du, Pearson, Hilmer, & Banks, 2017). Objective ascertainment of prescribed medication is likely to be provided in the future by linkage to primary care data, and in some studies, pharmacy claims data have been successfully used to supplement self‐reported medication (Drieling et al., 2016; Gnjidic et al., 2017). However, there will remain the likelihood that medication will have poor sensitivity for case finding in mental health, as psychotropics will never be prescribed to all of those with a lifetime history, and poor specificity as they are prescribed for many things outside of mental health. In the case of using medication in the UKB to supplement MHQ findings, there is the added problem of the snapshot of medication taken being ascertained around 7 years prior to the MHQ administration and therefore being unable to reflect new‐onset disorders and prescriptions.

Algorithmic approaches can be taken that exploit the strengths of each measure to produce a compound measure. Algorithms will include combining cases from two or more outcome types as done for this genomic study of depression in UKB using baseline self‐report and hospital diagnosis (Howard et al., 2018). Items can also be grouped into new criteria as was done to define mood disorders at baseline (Smith et al., 2013). Another approach, previously suggested in the case–control definitions defined by the UKB mental health outcomes group, uses symptom‐based outcomes for cases but excludes from controls those who self‐reported diagnosis or had data‐linkage diagnosis or suggestive medication. Taking the BPAD row from Table 4 as a simplified example: 2,247 people were positive for the symptom‐based outcome, and 155,119 were negative; out of those who did not meet criteria, 177 had a hospital diagnosis of BPAD, 326 more reported a diagnosis of BPAD, and 35 more reported taking lithium (Table 5c)—all of these are suggestive of BPAD. To minimise false positives and false negatives in the BPAD item, these 538 suggestive participants can be excluded from cases and controls, leaving 2,247 cases and 154,581 controls. Further algorithms incorporating hospital and primary care data for severe mental illness and common mental disorder in the full cohort are due to be published by UKB in 2019–2020—as has already been done for stroke and myocardial infarction.

### 4.2 Does it matter?

We have shown that different methods of ascertainment of mental disorder can result in different groups of participants being identified as cases. This poor agreement between methods of ascertainment could be problematic for research consistency and reproducibility. However, there is evidence that even with poor agreement at the level of disorder diagnosis, there can be similarity at the biological level. For example, a twin study (Torvik et al., 2018) reported that cases derived from interview diagnoses had limited overlap with those selected by data linkage (primary and secondary)—for depression, 36% interview positive were also on primary care registry, whereas 48% of those in registry were interview positive, with less overlap for anxiety (21%/46%) and alcohol use disorder (3%/33%). Despite this, the genetic features identified in the interview and registry groups were highly correlated within each diagnosis, approaching unity for depression and anxiety disorders. It remains to be seen whether the same will be true for the different cohorts selected in UKB—certainly focussing exclusively on very highly selected outcomes such as hospital data‐linkage means including biases to do with health service utilisation that may not relate to underlying mental health need (Roberts et al., 2018).

Genome‐wide association studies (GWASs) often pool cases and controls from different cohorts. Studies that define DSM disorders using clinical interview, self‐report diagnosis, symptom‐based outcomes, or combinations thereof might be combined in order to achieve the necessary size of the sample. The results will then depend heavily on whether the biology converges on a single disorder or converges on the different definitions (Vrieze, Iacono, & McGue, 2012). A massed GWAS of depression (Wray et al., 2018) included cases that were defined at interview (PGC29, GenScot), treatment registers (iPSYCHE, GERA), self‐report diagnosis (23andMe), and a combination (DeCODE, UKB [prior to MHQ results]) showed strong genetic correlation between the studies. The combined GWAS also showed enrichment of the targets of antidepressant treatment. These results suggest that weakening the phenotype can reveal interesting and relevant biology.

On some occasions, we have found that different measures have indicated different disorders for the same individuals, which could lead to confusion in research concentrated on a narrowly defined diagnosis. However, this reflects established findings of a high degree of co‐morbidity and cross‐over in mental disorders (Davis, Bashford, et al., 2018; First, 2005; Gask et al., 2008), probably due to shared aetiology and pathology (Cross‐Disorder Group of the Psychiatric Genomics Consortium, 2013; Elliott, Romer, Knodt, & Hariri, 2018) that is poorly translated into categorical diagnostic classifications. Other models for understanding mental disorder have been suggested, and some of these ideas could be translated to measures for research in large cohorts (Carcone & Ruocco, 2017; Clark et al., 2017; Vrieze et al., 2012), but diagnostic categories continue to be utilised widely.

### 4.3 Implications

For users of UKB, the symptom‐based outcomes defined in the MHQ offer advantages: They will select a large proportion of the participants with a likely disorder; many have been validated externally; and there is scope to customise, such as for different thresholds. However, self‐report, hospital data linkage, and medication may also be able to identify unique cases and may have high predictive validity. In some scenarios, it would seem sensible to add cases together. Another approach is to use the symptom‐based outcome to define the cases and define the controls to exclude positives on the other measures. For some questions, the sample and measures in the MHQ may be too limiting, and unprompted baseline self‐report supplemented by hospital data linkage will have to be used (Howard et al., 2018), which are highly selected, until primary care data and algorithms are released. Co‐morbidity between mental disorders is high, and interpretation of this may need consideration. Given the high degree of flexibility that UKB affords, researchers should consider the breadth and granularity of the mental health diagnosis needed alongside the consideration of the variables used to define them, so that the most appropriate combination of measure and outcome can be chosen to best address the research question.

Other studies could learn from the experience in UKB in three main ways. First, under‐recognition, fluctuating course, and self‐management of most mental disorder means questions about lifetime symptoms are needed to identify those who have never had a disorder. Second, co‐morbidity between the mental disorders is high, and this needs to be acknowledged in the design and interpretation of MHQs. Third, registries, data linkage, and measures of treatment will underestimate numbers of cases of mental disorder but do provide further information.

### 4.4 Strengths and weaknesses

UKB aims to produce and adjudicate outcomes in a clear, expert‐led manner. The Mental Health Outcomes Consortium has worked with UKB to implement the MHQ, and the present analysis was planned to clarify the different mental health definitions now present in UKB.

The MHQ had a very good response rate compared with previous UKB online questionnaires, and it gives an unparalleled sample size for a mental health survey. However, like much observational research, it is subject to participation bias in its volunteers (Davis, Coleman, et al., 2018; Fry et al., 2017). Given that participation in research can be patterned by mental health (Atherton, Fuller, Shepherd, Strachan, & Power, 2008; Knudsen, Hotopf, Skogen, Øverland, & Mykletun, 2010), it may be that people with severe symptoms of mental disorder were less likely to volunteer or complete the MHQ, as might be suggested by the small number of people with a hospital data‐linkage diagnosis of a psychotic disorder, which may limit generalisability of our findings to other settings.

The measures in the MHQ were felt to be the most suitable for defining lifetime mental disorders within the constraints of a short survey and maintaining compatibility with existing genetic studies. The online CIDI‐SF has been validated, but only for depression in the lifetime version. The questions used to assess for symptoms of mania/hypomania have not been externally validated. For both instruments, it is likely that the lifetime version is affected by recall bias. Further, the UKB data linkage and medication aspects are currently limited. Hospital admission data will capture few with mental disorders, so we will welcome the forthcoming linkages to primary care data. Medication was self‐reported and on a single occasion that was 7 to 10 years prior to the symptom‐based outcome: Again, it may be better after linkage to primary care data.

## 5 CONCLUSIONS

Large cohort studies provide great potential for interesting discovery, but using these datasets involves confronting problems with definitions of disorders, data quality, and incomplete coverage. Mental health research is further hampered the challenge that many mental disorders are under‐recognised and under‐represented in health care data. UKB is a rich observational resource due to its size, extensive baseline measures, and linkages to national administrative records. The utility of UKB for mental health research has been improved by the UKB MHQ. We have shown that, in general, the number of cases identified by lifetime symptom‐based diagnosis exceeds those identified with self‐report diagnosis, hospital data linkage, and psychotropic medication, with an overlap between measures that differs between the disorders under study. The advantage of symptom‐based lifetime classification of mental disorder is sensitivity across the severity spectrum, and many of the symptom‐based outcomes have been validated against psychiatric interview elsewhere. However, other mental health ascertainment methods could complement symptom‐based outcome measures in research. UKB and other open science projects lend themselves to innovative, well‐described, and reported approaches that can be scrutinised by the community. The ideas and results of this exploratory analysis highlight the strengths and limitations of both the indicators in large cohort studies, and the mental disorder diagnosis itself, which we hope will assist those planning to address the important questions in mental health and wider research.

## FUNDING INFORMATION

M. A. and A. M. are supported by a Wellcome Trust Strategic Award (Reference 104036/Z/14/Z). A. M. is supported by an MRC Grant (Reference MC_PC_17209) and The Sackler Trust. B. C. is funded by the Scottish Executive Chief Scientist Office (DTF/14/03) and by The Dr Mortimer and Theresa Sackler Foundation. W. L. is supported by the National Institute for Health Research (NIHR) Collaboration for Leadership in Applied Health Research and Care South West Peninsula.

## DECLARATION OF INTEREST STATEMENT

We have read and understood the author guidelines of ethical conduct and wish to declare the following: B. C. reports grants from the Scottish Executive Chief Scientist Office during the conduct of the study. M. H. reports grants for IMI RADAR‐CNS and personal fees as an expert witness outside the submitted work. G. B. reports grants from National Institute for Health Research during the conduct of the study and support from Illumina Ltd. and the European Commission outside the submitted work.

## Supporting information

Table S1: Overlap of routine items for common mental disorder and symptom‐based outcome for common mental disorder
Appendix S1: Questionnaire wording and format
Appendix S2: Case Criteria Derived from the UK Biobank Mental Health Questionnaire
Appendix S3: ICD‐10 codes used for hospital data‐linkage
Appendix S4: UKB medication codes used
