# Supplementary material for: Indicators of mental disorders in UK Biobank—A comparison of approaches
Source: Int J Methods Psychiatr Res. 2019 Aug 8;28(3):e1796. doi: 10.1002/mpr.1796 (PMC6877131; doi:10.1002/mpr.1796)
Supplement: Supplementary file 1 — Table S1: Overlap of routine items for common mental disorder and symptom‐based outcome for common mental disorder Appendix S1: Questionnaire wording and format Appendix S2: Case Criteria Derived from the UK Biobank Mental Health Questionnaire Appendix S3: ICD‐10 codes used for hospital data‐linkage Appendix S4: UKB medication codes used [file MPR-28-e1796-s001.docx]

Indicators of Mental Disorders in UK Biobank – A comparison of approaches. Davis et al., on behalf of the UK Biobank Mental Health Outcomes Consortium

**Supplementary Material**

Table SM1: [Overlap of routine items for common mental disorder and symptom-based outcome for common mental disorder](#SM1)

[Appendix 1: Questionnaire wording and format](#_Appendix_1:)

[Appendix 2: Case Criteria Derived from the UK Biobank Mental Health Questionnaire](#_Appendix_2:_Case)

[Appendix 3: ICD-10 codes used for hospital data-linkage](#_Appendix_3_ICD-10)

[Appendix 4: UKB medication codes used](#_Appendix_4_UKB)

**Table SM1: Overlap of routine items for common mental disorder and symptom-based outcome for common mental disorder**

Combinations of self-report clinician diagnosis of depression or anxiety (SR), hospital data-linkage (registry) of depression or anxiety, and antidepressant at baseline (meds) against symptom based outcomes of lifetime depression and generalised anxiety disorder, based on CIDI-SF, ordered by agreement by kappa.

| **Item(s)** | **n.** | **Symptom-based outcome**  **given**  **Item positive** | **Item positive**  **given**  **Symptom-based outcome** | **kappa** |
| --- | --- | --- | --- | --- |
| Registry | 4160 | 2811/4160 (68%) | 2811/37629 (7%) | 0.088 |
| Meds | 8616 | 5684/8616 (66%) | 5684/37629 (15%) | 0.166 |
| Registry or meds | 11009 | 7110/11009 (65%) | 7110/37629 (19%) | 0.199 |
| SR, registry or meds | 47321 | 25920/47321 (55%) | 25920/37629 (69%) | 0.454 |
| SR or meds | 46815 | 25803/46815 (55%) | 25803/37629 (69%) | 0.457 |
| SR or registry | 46153 | 25675/46153 (56%) | 25675/37629 (68%) | 0.461 |
| SR | 45585 | 25534/45585 (56%) | 25534/37629 (68%) | 0.463 |

Comparing symptom-based outcome with other reports for common mental disorders, agreement is highest between symptom-based outcome and self-report diagnosis (kappa=0.46). Adding registry diagnosis and/or self-reported medication data to self-report of common mental disorder identified a few extra participants (up to 1,736), but slightly reduced the level of agreement with the symptom-based outcome.

## Appendix 1: Questionnaire wording and format

Introduction

Section A: presence and absence of any mental health condition.

Section B: present and past depression and/or bipolar affective disorder.

Section C: generalised anxiety disorder.

Section D: addictions.

Section E: alcohol and cannabis use.

Section F: unusual and psychotic experiences.

Section G: events in childhood or adult life.

Section H: harm behaviours.

Section J: subjective wellbeing.

End page provides links to support.

| **Q.No** | **Field ID** | **Stem** | **Responses** |
| --- | --- | --- | --- |
| **Introduction** |  |  |  |
| **INTRO1** |  | We are interested in knowing more about the stresses and strains of life and your mental health. We realise that some of the questions are sensitive and may be difficult to answer but we hope you will feel able to take part. Participating in this questionnaire will help us understand mental health problems like depression and post traumatic stress disorder better. It’s important we hear from people who have had these sorts of problems as well as people who have not.  Your answers will be kept confidential. None of the information you provide will be sent to your GP or any other agencies.  We will not act on any concerns you raise in this questionnaire, and are not able to offer you any help based on the answers you give. However, information on where to find help for the issues in this questionnaire will be shown at the end.  Do not worry if you cannot answer a question – you can always press the “prefer not to answer” option and move on. | - Next |
| **Start1** |  | To help you as you work your way through the questionnaire:   - Most devices will allow you to click or tap the description beside a choice button to select it   **Click or tap on this sentence**  **Click /tap here**  **and over here**   - The progress bar at the bottom of each page (see below) is split into blocks, with each block representing a different section of the questionnaire. | - Next |
| **Identity check** |  | First, we need to check a couple of things. |  |
| **ID1**  **[no number on implementation]** |  | Please confirm your month and year of birth | mmm yyyy |
| **ID2**  **[no number on implementation]** |  | Please confirm your sex | [Select one from]  - 01 Male  - 02 Female |
| **INTRO2** |  | Now let’s start with a few general questions about mental distress. |  |
| **INTRO2restart** |  | Now let’s continue from where you left off… |  |
| **Section A: presence and absence of any mental health condition.** |  |  |  |
| **A1** | **20500** | In your life, have you suffered from a period of mental distress that prevented you from doing your usual activities? | [Select one from]  - 01 Yes  - 02 No  - UN Do not know  - DA Prefer not to answer |
| **A2** | **20499** | In your life, did you seek or receive help from a professional (medical doctor, psychologist, social worker, counsellor, nurse, clergy, or other helping professional) for mental distress, psychological problems or unusual experiences? | [Select one from]  - 01 Yes  - 02 No  - UN Do not know  - DA Prefer not to answer |
| **A3** | **20544** | Have you been diagnosed with one or more of the following mental health problems by a professional, even if you don’t have it currently? (tick all that apply):  By professional we mean: any doctor, nurse or person with specialist training (such as a psychologist or therapist). Please include disorders even if you did not need treatment for them or if you did not agree with the diagnosis. | [Select up to seven from]  - 01 Depression  - 02 Mania, hypomania, bipolar or manic-depression  - 03 Anxiety, nerves or generalized anxiety disorder  - 04 Social anxiety or social phobia  - 05 Agoraphobia  - 06 Any other phobia (eg disabling fear of heights or spiders  - 07 Panic attacks  - 08 Obsessive compulsive disorder (OCD)  - 00 None of the above  DA Prefer not to answer |
| **A4** | **20544** | Have you been diagnosed with one or more of the following~~;~~ mental health problems by a professional, even if you don’t have it currently? (tick all that apply):  By professional we mean: any doctor, nurse or person with specialist training (such as a psychologist or therapist). Please include disorders even if you did not need treatment for them or if you did not agree with the diagnosis. | [Select up to eight from]  - 01 Anorexia nervosa  - 02 Bulimia nervosa  - 03 Psychological over-eating or binge-eating  - 04 Schizophrenia  - 05 Any other type of psychosis or psychotic illness  - 06 A personality disorder  - 07 Autism, Asperger’s or autistic spectrum disorder  08 Attention deficit or attention deficit and hyperactivity disorder (ADD/ADHD)  - 00 None of the above  DA Prefer not to answer |
| **Section B: present and past depression and/or bipolar affective disorder.** |  |  |  |
| **INTRO3** |  | **We next want to ask a few questions about your mood and feelings recently:** | - Next |
| **B1** | 1. **20514** 2. **20510** 3. **20534** 4. **20519** 5. **20511** 6. **20507** 7. **20508** 8. **20518** 9. **20513** | Over the last 2 weeks, how often have you been bothered by any of the following problems?  a. Little interest or pleasure in doing things  b. Feeling down, depressed, or hopeless  c. Trouble falling or staying asleep, or  sleeping too much  d. Feeling tired or having little energy  e. Poor appetite or overeating  f. Feeling bad about yourself or that you  are a failure or have let yourself or your  family down  g. Trouble concentrating on things, such  as reading the newspaper or watching  television  h. Moving or speaking so slowly that other  people could have noticed? Or the opposite — being so fidgety or restless that you have been moving around a lot more than usual  i. Thoughts that you would be better off  dead or of hurting yourself in some way | [Select one from the following for each of the statements]  - 01 Not at all  - 02 Several days  - 03 More than half the days  - 04 Nearly every day  - DA Prefer not to answer |
| **BSTEM1** |  | **Now we want to know some more about symptoms in your lifetime** | |
| **B2** | **20446** | Have you ever had a time in your life when you felt sad, blue, or depressed for two weeks or more in a row? | [Select one from]  - 01 Yes  - 00 No  - DA Prefer not to answer |
| **B3** | **20441** | Have you ever had a time in your life lasting two weeks or more when you lost interest in most things like hobbies, work, or activities that usually give you pleasure? | [Select one from]  - 01 Yes  - 00 No  - DA Prefer not to answer |
| **BSTEM2** |  | Please think of the two-week period in your life when your feelings of depression or loss of interest were worst: | Display throughout following questions B4 to B14 |
| **B4** | **20447** | Did this worst period start within two months of the death of someone close to you or after a stressful or traumatic event in your life? | [Select one from]  - 01 Yes  - 00 No  - DA Prefer not to answer |
| **B5** | **20436 (Fraction of day affected)** | How much of the day did these feelings usually last? | - 04 All day long  - 03 Most of the day  - 02 About half of the day  - 01 Less than half of the day  - NA Do not know  - DA Prefer not to answer |
| **B6** | **20439 (Frequency of depressed days)** | Did you feel this way | - 03 Every day  - 02 Almost every day  - 01 Less often  - NA Do not know  - DA Prefer not to answer |
| **B7** | **20449** | Did you feel more tired out or low on energy than is usual for you? | - 01 Yes  - 00 No  - NA Do not know  - DA Prefer not to answer |
| **B8** | **20536** | Did you gain or lose weight without trying, or did you stay about the same weight? | - 01 Gained weight  - 02 Lost weight  - 03 Both gained and lost some weight during the episode  - 00 Stayed about the same or was on a diet  - NA Do not know  - DA Prefer not to answer |
| **B9** | **20532** | Did your sleep change? | - 01 Yes  - 00 No  - NA Do not know  - DA Prefer not to answer |
| **B9a** | **a) 20533**  **b) 20535**  **c) 20534** | Was that:  a Trouble falling asleep  b Waking too early  c Sleeping too much | [Three questions grouped together, each with forced choice]  - 01 Yes  - 00 No |
| **B10** | **20435** | Did you have a lot more trouble concentrating than usual? | - 01 Yes  - 00 No  - NA Do not know  - DA Prefer not to answer |
| **B11** | **20450** | People sometimes feel down on themselves, no good, worthless. Did you feel this way? | - 01 Yes  - 00 No  - NA Do not know  - DA Prefer not to answer |
| **B12** | **20437** | Did you think a lot about death – either your own, someone else’s or death in general? | - 01 Yes  - 00 No  - UN Do not know  - DA Prefer not to answer |
| **B13** | **20438 (Duration of worst depression)** | About how long altogether did you feel this way? Count the time before, during and after the worst two weeks. | - 01 Less than a month  - 02 Between one and three months  - 03 Over three months, but less than six months  - 04 Over six months, but less than 12 months  - 05 One to two years  - 06 Over two years  - DA Prefer not to answer |
| **B14** | **20440** | Think about your roles at the time of this episode, including study / employment, childcare and housework, leisure pursuits. How much did these problems interfere with your life or activities? | - 03 A lot  - 02 Somewhat  - 01 A little  - 00 Not at all  - DA Prefer not to answer |
| **BSTEM3** |  | Regarding times in your life when you have had feelings of depression or loss of interest: | Display throughout following questions B15 to B20 |
| **B15** | **20442** | How many periods did you have in your life lasting two or more weeks where you felt like this? | - 01 One  - 02 Several  - DA Prefer not to answer |
| **B15a** | **20442** | Enter number | BBOX1: Integer box 2 – 999  BBOX1 & “number of times”  OR  - 01 Too many to count / One episode ran into the next. |
| **B16** | **20433** | About how old were you the FIRST time you had a period of two weeks like this? (Whether or not you received any help for it.) | BBOX2: Integer box 2 to current age  BBOX2 & “years of age when first felt this way”  OR  - UN Do not know  OR  - DA Prefer not to answer |
| **B17** | **20445** | Did this episode occur within months of giving birth? Or has it been suggested you had post-natal depression? | - 01 Yes  - 00 No  - NA Not applicable  - UN Do not know  - DA Prefer not to answer |
| **B18** | **20434** | About how old were you the LAST time you had a period of two weeks like this? (Whether or not you received any help for it) | BBOX3: Integer box 2 to current age  BBOX3 & “years of age when last felt this way”  Or  - UN Don’t know  Or  - DA Prefer not to answer |
| **B19** | **20448** | Did you ever tell a professional about these problems (medical doctor, psychologist, social worker, counsellor, nurse, clergy, or other helping professional)? | - 01 Yes  - 00 No  - UN Do not know  - DA Prefer not to answer |
| **B20** | **20546** | Did you ever try the following for these problems? (tick all that apply) | [Select up to three]  - 01 Medication prescribed to you (for at least two weeks)  - 02 Unprescribed medication  (more than once)  - 03 Drugs or alcohol (more than once)  - 00 None of the above  - DA Prefer not to say |
| **B21** | **20547** | Did you ever try talking therapies for these problems, or other structured activities you regard as therapeutic? Include only those you attended more than once. | [Select up to two]  - 01 Talking therapies, such as psychotherapy, counselling, group therapy or CBT  - 02 Other therapeutic activities such as mindfulness, yoga or art classes  - 00 None of the above  - DA Prefer not to answer |
| **BSTEM4** |  | Now we want to know about some different symptoms. | - Next |
| **B22** | **20501** | Have you ever had a period of time when you were feeling so good, “high”, “excited”, or “hyper” that other people thought you were not your normal self or you were so “hyper” that you got into trouble? | - 01 Yes  - 00 No  - UN Do not know  - DA Prefer not to answer |
| **B23** | **20502** | Have you ever had a period of time when you were so irritable that you found yourself shouting at people or starting fights or arguments? | - 01 Yes  - 00 No  - UN Do not know  - DA Prefer not to answer |
| **B24** | **20548** | Please try to remember a period when you were in a “high” or “irritable” state and select all of the following that apply: | [Select up to eight]  - 01 I was more active than usual  - 02 I was more talkative than usual  - 03 I needed less sleep than usual  - 04 I was more creative or had more ideas than usual  -05 I was more restless than usual  -06 I was more confident than usual  - 07 My thoughts were racing  - 08 I was easily distracted  - 00 None of the above  - DA Prefer not to answer |
| **B25** | **20492** | What is the longest time that these “high” or “irritable” periods have lasted? | [Choose one of]  - 01 Less than 24 hours  - 02 At least a day, but less than a week  - 03 A week or more  - UN Do not know  - DA Prefer not to answer |
| **B26** | **20493** | How much of a problem have these “high” or “irritable” periods caused you? | - 00 No problems  - 01 Needed treatment or caused problems with work, relationships, finances, the law or other aspects of life.  - UN Do not know  - DA Prefer not to say |
| **Section C: generalised anxiety disorder.** |  |  |  |
| **INTRO4** |  | We want to know some more about anxiety | Next |
| **C1** | 1. **20506** 2. **20509** 3. **20520** 4. **20515** 5. **20516** 6. **20505** 7. **20512** | Over the last 2 weeks, how often have you been bothered by any of the following problems?  a) Feeling nervous, anxious or on edge  b) Not being able to stop or control worrying  c) Worrying too much about different things  d) Trouble relaxing  e) Being so restless that it is hard to sit still  f) Becoming easily annoyed or irritable  g) Feeling afraid as if something awful might happen  [7 questions on one screen in grid] | [Select one from the following for each of the statements]  - 01 Not at all  - 02 Several days  - 03 More than half the days  - 04 Nearly every day  - DA Prefer not to answer |
| **C2** | **20421** | Have you ever had a period lasting one month or longer when most of the time you felt worried, tense, or anxious? | - 01 Yes  - 00 No  - UN Do not know  - DA Prefer not to answer |
| **C2a** | **20420** | What is the longest period of time that this kind of worrying has ever continued? | Cbox2: Integer 0-99  Cbox1: Integer 0-11  Cbox02 & “year(s) and” & Cbox01 & “month(s)”  OR  - 03 All my life / as long as I can remember |
| **C3** | **20425** | People differ a lot in how much they worry about things. Did you ever have a time when you worried a lot more than most people would in your situation? | - 01 Yes  - 00 No  - UN Do not know  - DA Prefer not to answer |
| **CSTEM1** |  | Please think of the period in your life when you have felt worried, tense, anxious, or more worried than most people would in your situation. This could be in the past, or it could be continuing now. | Display throughout following questions C4 to C10 |
| **C4** | **20542** | During that period, was your worry stronger than in other people? | - 01 Yes  - 00 No  - UN Do not know  - DA Prefer not to answer |
| **C5** | **20538** | Did you worry most days? | - 01 Yes  - 00 No  - UN Do not know  - DA Prefer not to answer |
| **C6** | **20543** | Did you usually worry about one particular thing, such as your job security or the failing health of a loved one, or more than one thing? | - 01 One thing  - 02 More than one thing  - UN Do not know  - DA Prefer not to answer |
| **C7** | **20541** | Did you find it difficult to stop worrying? | - 01 Yes  - 00 No  - UN Do not know  - DA Prefer not to answer |
| **C8** | **20540** | Did you ever have different worries on your mind at the same time? | - 01 Yes  - 00 No  - UN Do not know  - DA Prefer not to answer |
| **C9** | **20539** | How often was your worry so strong that you couldn’t put it out of your mind no matter how hard you tried? | - 03 Often  - 02 Sometimes  - 01 Rarely  - 00 Never  - UN Do not know  - DA Prefer not to answer |
| **C10** | **20537** | How often did you find it difficult to control your worry? | - 03 Often  - 02 Sometimes  - 01 Rarely  - 00 Never  - UN Do not know  - DA Prefer not to answer |
| **C11** | 1. **20426** 2. **20423** 3. **20429** 4. **20419** 5. **20422** 6. **20417** 7. **20427** | When you were worried or anxious, were you also:  a) Restless?  b) Keyed up or on edge?  c) Easily tired?  d) Having difficulty keeping your mind on what you were doing?  e) More irritable than usual?  f) Having tense, sore, or aching muscles?  g) Often having trouble falling or staying asleep?  [Seven questions on one screen] | Force choice:  - 01 Yes  - 02 No  - NA Do not know  For following options: |
| **CSTEM2** |  | Regarding times in your life when you have felt worried, tense or anxious: | Display throughout following questions C12 to C15 |
| **C12** | **20428** | Did you ever tell a professional about these problems (medical doctor, psychologist, social worker, counsellor, nurse, clergy, or other helping professional)? | - 01 Yes  - 00 No  - UN Do not know  - DA Prefer not to answer |
| **C13** | **20549** | Did you ever use the following for the worry or the problems it caused? (tick all that apply):  Please include any treatments that you have already told us about under ‘depression’ if they were also for anxiety: | - 01 Medication prescribed to you (for at least two weeks)  - 02 Unprescribed medication  (more than once)  - 03 Drugs or alcohol (more than once)  - 00 None of the above  - DA Prefer not to say |
| **C14** | **20550** | Did you ever try talking therapies for these problems, or other structured activities you regard as therapeutic? Include only those you attended more than once.  Please include any treatments that you have already told us about under “depression” if they were also for anxiety: | [Select up to two]  - 01 Talking therapies, such as psychotherapy, counselling, group therapy or CBT  - 02 Other therapeutic activities such as mindfulness, yoga or art classes  - 00 None of the above  - DA Prefer not to answer |
| **C15** | **20418** | Think about your roles at the time of this episode, including study / employment, childcare and housework, leisure pursuits. How much did these problems interfere with your life or activities? | [Choose one of]  - 03 A lot  - 02 Somewhat  - 01 A little  - 00 Not at all  - DA Prefer not to answer |
| **Section D: addictions.** |  |  |  |
| **INTRO5** |  | Now we’d like to ask you a few questions about addiction and dependence |  |
| **D1** | **20401** | Have you been addicted to or dependent on one or more things, including substances (not cigarettes/coffee) or behaviours (such as gambling)? | [Select one from]  - 01 Yes  - 00 No  - UN Do not know  - DA Prefer not to answer |
| **D2** | **20406** | Have you been addicted to alcohol? | [Select one from]  - 01 Yes  - 00 No  - UN Do not know  - DA Prefer not to answer |
| **D2a** | **20415** | Is this addiction ongoing? | [Select one from]  - 01 Yes  - 00 No  - DA Prefer not to answer |
| **D2b** | **20404** | Have you been physically dependent on alcohol?  This means experiencing withdrawal symptoms, such as sweating, shaking and nausea, if you didn’t drink. | [Select one from]  - 01 Yes  - 00 No  - UN Do not know  - DA Prefer not to answer |
| **D3** | **20503** | Have you been addicted to or dependent on prescription or over-the-counter medication? | [Select one from]  - 01 Yes  - 00 No  - UN Do not know  - DA Prefer not to answer |
| **D3a** | **20551** | Was this addiction or dependence to one of the following? (tick all that apply) | [Select up to three from]  - 01 A sedative, benzodiazepine or sleeping tablet  - 02 A painkiller  - 00 Something else  - UN Do not know  - DA Prefer not to answer |
| **D3b** | **20504** | Is this addiction or dependence ongoing? | [Select one from]  - 01 Yes  - 00 No  - DA Prefer not to answer |
| **D4** | **20456** | Have you been addicted to Illicit or recreational drugs? | [Select one from]  - 01 Yes  - 00 No  - UN Do not know  - DA Prefer not to answer |
| **D4a** | **20457** | Is this addiction or dependence ongoing? | [Select one from]  - 01 Yes  - 00 No  - DA Prefer not to answer |
| **D5** | **20431** | Have you been addicted to a behaviour (such as gambling) or to anything else we have not mentioned? | [Select one from]  - 01 Yes  - 00 No  - UN Do not know  - DA Prefer not to answer |
| **D5a** | **20552** | Were you addicted to: (tick all that apply) | [Select up to two from]  - 01 A behaviour  - 02 Something else not mentioned  - DA Prefer not to answer |
| **D5b** | **20432** | Are these addictions ongoing? | [Select one from]  - 01 Yes  - 00 No  - DA Prefer not to answer |
| **Section E: alcohol and cannabis use.** |  |  |  |
| **INTRO6** |  | Next we would like to ask you about alcohol, as we think it may influence mental health. Your answers will remain confidential so please be honest. | Next |
| **ESTEM1** |  | The next questions are about how frequently you drink alcohol. | Stay on screen for questions E1-E1b |
| **E1** | **20414** | How often do you have a drink containing alcohol? | [Choose one from]  - 00 Never  - 01 Monthly or less  - 02 2 to 4 times a month  - 03 2 to 3 times a week  - 04 4 or more times a week  - DA Prefer not to answer |
| **ESTEM2** |  | \| In the next two questions, a "drink" is defined as one unit of alcohol.  Typical units in common alcoholic beverages \| \| \| \| \| --- \| --- \| --- \| --- \| \| 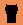 \| Pint or can of beer/lager/cider \| 2 units \| \| 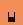 \| Single shot of spirits (25ml) \| 1 unit \| \| 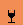 \| Small glass of fortified wine \| 1 unit \| \| 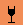 \| Standard glass of wine (175ml) \| 2 units \| \| 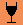 \| Large glass of wine (250ml) \| 3 units \| \| 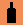 \| Bottle of wine (75cl) \| 9 units \| | Stay on screen for questions E1a and E1b |
| **E1A** | **20403** | How many drinks containing alcohol do you have on a typical day when you are drinking?  By “drink” we mean one unit of alcohol. | [Choose one from]  - 01 1 or 2  - 02 3 or 4  - 03 5 or 6  - 04 7, 8, or 9  - 05 10 or more  - DA Prefer not to answer |
| **E1B** | **20416** | How often do you have six or more drinks on one occasion?  By “drink” we mean one unit of alcohol. | [Choose one from]  - 01 Never  - 02 Less than monthly  - 03 Monthly  - 04 Weekly  - 05 Daily or almost daily  - DA Prefer not to answer |
| **E2** | **20413** | How often during the last year have you found that you were not able to stop drinking once you had started? | [Choose one from]  - 00 Never  - 01 Less than monthly  - 02 Monthly  - 03 Weekly  - 04 Daily or almost daily  - DA Prefer not to answer |
| **E3** | **20407** | How often during the last year have you failed to do what was normally expected from you because of drinking? | [Choose one from]  - 01 Never  - 02 Less than monthly  - 03 Monthly  - 04 Weekly  - 05 Daily or almost daily  - DA Prefer not to answer |
| **E4** | **20412** | How often during the last year have you needed a first drink in the morning to get yourself going after a heavy drinking session? | [Choose one from]  - 01 Never  - 02 Less than monthly  - 03 Monthly  - 04 Weekly  - 05 Daily or almost daily  - DA Prefer not to answer |
| **E5** | **20409** | How often during the last year have you had a feeling of guilt or remorse after drinking? | [Choose from]  - 01 Never  - 02 Less than monthly  - 03 Monthly  - 04 Weekly  - 05 Daily or almost daily  - DA Prefer not to answer |
| **E6** | **20408** | How often during the last year have you been unable to remember what happened the night before because you had been drinking? | [Choose one from]  - 01 Never  - 02 Less than monthly  - 03 Monthly  - 04 Weekly  - 05 Daily or almost daily  - DA Prefer not to answer |
| **E7** | **20411** | Have you or someone else been injured as a result of your drinking? | [Choose one from]  - 00 No  -01 Yes, but not in the last year  - 02 Yes, during the last year  - DA Prefer not to answer |
| **E8** | **20405** | Has a relative or friend or a doctor or another health worker been concerned about your drinking or suggested you cut down? | [Choose one from]  - 00 No  - 01 Yes, but not in the last year  - 02 Yes, during the last year  - DA Prefer not to answer |
| **E8a** | **20410** | About how old were you when somebody last commented this way about your drinking habits? | DBOX1: Integer box 2 to current age  DBOX1 & “years old”  Or  - NA Do not know  - DA Prefer not to answer |
| **ESTEM3** |  | Now we’d like to ask you about cannabis. Your answers will remain confidential |  |
| **E9** | **20453** | Have you taken CANNABIS (marijuana, grass, hash, ganja, blow, draw, skunk, weed, spliff, dope), even if it was a long time ago? | [Choose one from]  - 00 No  - 01 Yes, 1-2 times  - 02 Yes, 3-10 times  - 03 Yes, 11-100 times  -04 Yes, more than 100 times  - DA Prefer not to answer |
| **E9a** | **20454** | Considering when you were taking cannabis most regularly, how often did you take it? | [Choose one from]  - 01. Less than once a month  - 02. Once a month or more, but not every week  - 03. Once a week or more, but not every day  - 04. Every day  - NA Do not know  - DA Prefer not to answer |
| **E9b** | **20455** | About how old were you when you last had cannabis? | EBox1: Integer box 2 to current age  EBox1 & “years old” |
| **Section F: unusual and psychotic experiences.** |  |  |  |
| **INTRO7** |  | The next set of questions is about unusual experiences that you may have had, like seeing visions or hearing voices. We believe that these things may be quite common, but we don't know for sure. So please take your time and think carefully before answering. |  |
| **F1** | **20471** | Did you ever see something that wasn’t really there that other people could not see?  Please do not include any times when you were dreaming or half-asleep or under the influence of alcohol or drugs. | [Choose one from]  - 01 Yes  - 00 No  - NA Do not know  - DA Prefer not to answer |
| **F1a** | **20473** | About how many times in your life did this happen (when you were not dreaming, not half-asleep, and not under the influence of alcohol or drugs)? | FBOX1: Integer box 1 – 999  FBOX1 & “time(s)”  OR  - 01 Too many to count  - NA Do not know  - DA Prefer not to answer |
| **F2** | **20463** | Did you ever hear things that other people said did not exist, like strange voices coming from inside your head talking to you or about you, or voices coming out of the air when there was no one around?  Please do not include any times when you were dreaming or half-asleep or under the influence of alcohol or drugs. | [Choose one from]  - 01 Yes  - 00 No  - DA Prefer not to say  - NA Don’t know |
| **F2a** | **20465** | About how many times in your life did this happen (when you were not dreaming, not half-asleep, and not under the influence of alcohol or drugs)? | FBOX2: Integer box 1 – 999  FBOX2 & “time(s)”  OR  - 01 Too many to count  - NA Do not know  - DA Prefer not to answer |
| **F3** | **20474** | Did you ever believe that a strange force was trying to communicate directly with you by sending special signs or signals that you could understand but that no one else could understand (for example through the radio or television)?  Please do not include any times when you were dreaming or half-asleep or under the influence of alcohol or drugs. | [Choose one from]  - 01 Yes  - 00 No  - NA Do not know  - DA Prefer not to answer |
| **F3a** | **20476** | About how many times in your life did this happen (when you were not dreaming, not half-asleep, and not under the influence of alcohol or drugs)? | FBOX3: Integer box 1 – 999  FBOX3 & “time(s)”  OR  - 01 Too many to count  - NA Do not know  - DA Prefer not to answer |
| **F4** | **20468** | Did you ever believe that that there was an unjust plot going on to harm you or to have people follow you, and which your family and friends did not believe existed?  Please do not include any times when you were dreaming or half-asleep or under the influence of alcohol or drugs. | [Choose one from]  - 01 Yes  - 00 No  - NA Do not know  - DA Prefer not to answer |
| **F4a** | **20470** | About how many times in your life did this happen (when you were not dreaming, not half-asleep, and not under the influence of alcohol or drugs)? | FBOX4: Integer box 1 – 999  FBOX4 & “time(s)”  OR  - 01 Too many to count  - NA Do not know  - DA Prefer not to answer |
| **F5** | **20467** | How often did any of these experiences happen in the past 1 year (seeing a vision, hearing a voice, or believing that something strange was trying to communicate with you, or there was a plot against you)? | [Choose one from]  - 00 Not at all  - 01 Once or twice  - 02 Less than once a month  - 03 More than once a month  - 04 Nearly every day or daily  - DA Prefer not to answer |
| **F6** | **20461** | How old were you (approximately) when you first had one of these experiences (seeing a vision, hearing a voice, or believing that something strange was trying to communicate with you, or there was a plot against you)? | FBOX5: Integer box 2 to current age  FBOX5 & “years old”  OR  - 01 As long as I can remember  - NA Do not know  - DA Prefer not to answer |
| **F7** | **20462** | How distressing did you find having any of these experiences (seeing a vision, hearing a voice, or believing that something strange was trying to communicate with you, or there was a plot against you)? | [Choose one from]  - 00 Not distressing at all, it was a positive experience  - 01 Not distressing, a neutral experience  - 02 A bit distressing  - 03 Quite distressing  - 04 Very distressing  - NA Do not know  - DA Prefer not to answer |
| **F8** | **20477** | Did you ever talk to a doctor, counsellor, psychiatrist or other health professional about any of these experiences (seeing a vision, hearing a voice, or believing that something strange was trying to communicate with you, or there was a plot against you)? | [Choose one from]  - 01 Yes  - 00 No  - NA Do not know  - DA Prefer not to answer |
| **F9** | **20466** | Were you ever prescribed a medication by a health professional for any of these experiences (seeing a vision, hearing a voice, or believing that something strange was trying to communicate with you, or there was a plot against you)? | [Choose one from]  - 01 Yes  - 00 No  - NA Do not know  - DA Prefer not to answer |
| **Section G: events in childhood or adult life.** |  |  |  |
| **INTRO8** |  | This section asks about your childhood and some possible stresses and strains of life. The answers you give will remain confidential. |  |
| **G1** | 1. **20489** 2. **20488** 3. **20487** 4. **20490** 5. **20491** | When I was growing up…  a) I felt loved  b) People in my family hit me so hard that it left me with bruises or marks  c) I felt that someone in my family hated me  d) Someone molested me (sexually)  e) There was someone to take me to the doctor if I needed it  [Five questions on one screen with same options.] | [Select one from]  - 00 Never true  - 01 Rarely true  - 02 Sometimes true  - 03 Often  - 04 Very often true  - DA Prefer not to answer` |
| **G2** | 1. **20522** 2. **20523** 3. **20521** 4. **20524** 5. **20525** | Since I was sixteen…  a) I have been in a confiding relationship  b) A partner or ex-partner deliberately hit me or used violence in any other way  c) A partner or ex-partner repeatedly belittled me to the extent that I felt worthless  d) A partner or ex-partner sexually interfered with me, or forced me to have sex against my wishes  e) There was money to pay the rent or mortgage when I needed it  [Five questions on one screen with same options.] | [Select one from]  - 00 Never true  - 01 Rarely true  - 02 Sometimes true  - 03 Often  - 04 Very often true  - DA Prefer not to answer |
| **G3** | 1. **20531** 2. **20529** 3. **20526** 4. **20530** 5. **20528** 6. **20527** | In your life, have you..?  a) Been a victim of a sexual assault, whether by a stranger or someone you knew  b) Been attacked, mugged, robbed, or been the victim of a physically violent crime  c) Been in a serious accident that you believed to be life-threatening at the time  d) Witnessed a sudden violent death (eg. murder, suicide, aftermath of an accident)  e) Been diagnosed with a life-threatening illness  f) Been involved in combat or exposed to a war-zone (either in the military or as a civilian)  [Six questions on one screen with the same options.] | [Select one from]  - 00 Never  - 01 Yes, but not in the last 12 months  - 02 Yes, within the last 12 months  - DA Prefer not to answer |
| **G4** | 1. **20497** 2. **20498** 3. **20495** | Next is a list of problems and complaints that people sometimes have in response to such extremely stressful experiences. Please indicate how much you have been bothered by that problem in the past month:  a) Repeated, disturbing memories, thoughts, or images of a stressful experience?  b) Feeling very upset when something reminded you of a stressful experience?  c) Avoiding activities or situations because they reminded you of a stressful experience?  [Three questions on the same screen with the options] | – [Choose one of]  - 00 Not at all  - 01 A little bit  - 02 Moderately  - 03 Quite a bit  - 04 Extremely  - DA Prefer not to answer |
| **G5** | 1. **20496** 2. **20494** | Please indicate how much you have been bothered by that problem in the past month:  a) Feeling distant or cut off from other people?  b) Feeling irritable or having angry outbursts?  [Two questions on the same screen with the options] | [Choose one of]  - 00 Not at all  - 01 A little bit  - 02 Moderately  - 03 Quite a bit  - 04 Extremely  - DA Prefer not to answer |
| **Section H: harm behaviours.** |  |  |  |
| **INTRO9** |  | This section is about thoughts that some people have when they are distressed. | - Next |
| **H1** | **20479** | Many people have thoughts that life is not worth living. Have you felt that way? | [Choose one of]  - 00 No  - 01 Yes, once  - 02 Yes, more than once  - DA Prefer not to answer |
| **H2** | **20485** | Have you contemplated harming yourself (for example by cutting, biting, hitting yourself or taking an overdose)? | [Choose one of]  - 00 No  - 01 Yes, once  - 02 Yes, more than once  - DA Prefer not to answer |
| **H2a** | **20486** | Have you felt this way in the last 12 months? | [Choose one of]  - 00 No  - 01 Yes  - DA Prefer not to answer |
| **H3** | **20480** | Have you deliberately harmed yourself, whether or not you meant to end your life? | [Choose one of]  - 00 No  - 01 Yes  - DA Prefer not to answer |
| **H3a** | **20482** | How many times have you harmed yourself? | [Choose one of]  - 01 1  - 02 2  - 03 3 or more  - DA Prefer not to answer |
| **H3b** | **20481** | Have you harmed yourself in the last 12 months, whether or not you meant to end your life? | [Choose one of]  - 00 No  - 01 Yes  - DA Prefer not to answer |
| **H4** | **20553** | Have you done any of the following to harm or endanger yourself? (tick all that apply) | [Choose up to five]  - 01 Self-injury such as self-cutting, scratching or hitting, etc.  - 02 Ingesting a medication in excess of the normal dose  - 03 Ingesting alcohol or a recreational or illicit drug  - 04 Swallowing dangerous objects or products  - 05 Stopping prescribed medication  - 00 something not listed  - DA Prefer not to answer |
| **H5** | **20483** | Have you harmed yourself with the intention to end your life? | [Choose one]  - 00 No  - 01 Yes  - DA Prefer not to answer |
| **H5a** | **20484** | Was this in the last 12 months? | [Choose one]  - 00 No  - 01 Yes  - DA Prefer not to answer |
| **H6** | **20554** | Following any time when you took an overdose or deliberately tried to harm yourself did you (tick all that apply) | [Choose up to five]  - 01 Need hospital treatment (eg A&E)?  - 02 See anyone from psychiatric or mental health services, including liaison services?  - 03 See your GP?  - 04 Receive help from friends / family / neighbours?  - 05 Use a helpline / voluntary organization?  - 00 None of the above  - DA Prefer not to answer |
| **Section J: subjective wellbeing.** |  |  |  |
| **INTRO10** |  | Finally we would like to know how you feel about things in general |  |
| **J1** | **20458** | In general how happy are you? | - 01 Extremely happy  - 02 Very happy  - 03 Moderately happy  - 04 Moderately unhappy  -05 Very unhappy  - 06 Extremely unhappy  - UN Do not know  - DA Prefer not to answer |
| **J2** | **20459** | In general how happy are you with your HEALTH? | - 01 Extremely happy  - 02 Very happy  - 03 Moderately happy  - 04 Moderately unhappy  -05 Very unhappy  - 06 Extremely unhappy  - UN Do not know  - DA Prefer not to answer |
| **J3** | **20460** | To what extent do you feel your life to be meaningful? | - 01 Not at all  - 02 A little  - 03 A moderate amount  - 04 Very much  - 05 An extreme amount  - UN Do not know  - DA Prefer not to answer |
| **J4** |  | Please use the space below to tell us any further information relevant to this questionnaire. Any information you provide here will not be made available to researchers for research purposes. Please remember that we will not action any concerns you raise in this questionnaire; details of possible sources of support are provided on the next screen [max 1000 characters].  Please note: after you press the Save and finish button below, you will no  longer be able to change your answers. | Text box: 1000 characters |
| Thank you very much for taking the time to complete this questionnaire. Your help is greatly appreciated.  If you feel you need any further help with the issues in this questionnaire, we recommend talking it through with someone you trust, including your GP.  You can find out more about mental health and illness from Mind ([www.mind.org.uk](http://www.mind.org.uk)). General tips to help you cope with everyday things like money, work, and more are available from: <http://www.mind.org.uk/information-support/tips-for-everyday-living/>  For support with specific issues, further information is available from:  [www.thesurvivorstrust.org](http://www.thesurvivorstrust.org) (sexual violence)  <https://www.victimsupport.org.uk/help-victims> (other crime and violence)  <https://www.drinkaware.co.uk> (alcohol)  <http://www.combatstress.org.uk/> (information for military veterans)  <http://www.mind.org.uk/news-campaigns/campaigns/bluelight/> (information for emergency service personnel)  <https://www.rnli.org/aboutus/lifeguardsandbeaches/Pages/volunteer-lifeguards/support-and-advice.aspx> (information for RNLI employees and volunteers).  If you are very upset or do not feel safe, please contact someone as soon as possible. The Samaritans can be contacted on Freephone 116 123, or email [jo@samaritans.org](mailto:jo@samaritans.org). Alternatively, please visit [www.samaritans.org](http://www.samaritans.org)  You may now close this browser tab, if you so wish. | | | |

## Appendix 2: Case Criteria Derived from the UK Biobank Mental Health Questionnaire

Definitions used in Davis et al., Indicators of MH are identified with a double star (**)

Control definitions are fairly stringent, creating control groups for applications where higher certainty that control groups do not contain cases.

| **Disorder / Exposure** | **Rule in English** | **Fields and codes** | **Notes and references** | |
| --- | --- | --- | --- | --- |
| Mood disorder | | | | |
| Depression  ** | **Case: Depression ever.**  At least one core symptom of depression, most or all of the day on most or all days for a two week period, with at least five depressive symptoms that represent a change from usual occurring over the same time-scale, with some or a lot of impairment. | Persistent sadness (20446) = Yes OR Loss of interest (20441) = Yes  AND  How much of day (20436) = Most of day or All day long  AND  Did you feel this way (20439) = Almost every day or Every day  AND  Impairment (20440) = Somewhat or A lot  AND  Total number of symptoms endorsed (core and others) >= 5   - Persistent sadness (core) 20446; Loss of interest (core) 20441; Tired or low energy 20449; Gain or loss of weight 20536 = Gain, Loss or Gain and loss; Sleep change 20532; Trouble concentrating 20435; Feeling worthless 20450; Thinking about death 20437 | CIDI-SF (Composite International Diagnostic Interview – Short Form), depression module, lifetime version. Scored based on DSM definition of major depressive disorder  *Kessler RC, Andrews G, Mroczek D, Ustun B, Wittchen HU. The World Health Organization composite international diagnostic interview short‐form (CIDI‐SF). Int J Methods Psychiatr Res. 1998;7(4):171-85.* |  |
| Depression | **Case: Subthreshold depressive symptoms ever.**  Does not meet diagnostic criteria for depression on the CIDI, but has at least one of: (i) endorses persistent depression or anhedonia on CIDI; (ii) PHQ9 (current depressive symptoms) is over threshold for mild depression; (iii) reports clinician diagnosis of depression | NOT Case {depression ever}  AND  ((reported diagnosis of depression 20544 or 20002)  OR  Core symptoms from above  OR  PHQ score >5) | Case plus control plus subthreshold should include all participants with valid responses  Subthreshold symptoms may have clinical significance  *National Institute for Health and Clinical Excellence. Depression in adults: recognition and management. NICE Clinical Guideline CG90 (available at https://www.nice.org.uk/guidance/cg90) 2009 (updated 2016).* |  |
| Depression | **Control: Depression ever.**  Not endorsing depression or screening positive on PHQ or CIDI | NOT (reported diagnosis of depression 20544 or 20002)  AND  NOT Core symptoms from above  AND  PHQ score ≤5 | Case plus control plus subthreshold should include all participants with valid responses. By excluding subthreshold symptoms, we can be confident that this group has not experienced a classical depressive episode |  |
| Depression | **Case: Depression single episode.** | Case {depression ever}  AND  Number of episodes (20442)=1  AND  NOT case {bipolar type I}  Excluded if number of episodes missing or bipolar state missing | Single episode, recurrent depression and bipolar type I should include all depression cases with valid responses |  |
| Depression | **Case: Recurrent depression.** | {depression ever}  AND  Number of episodes (20442) >1 or -999 (too many to count)  AND  NOT case {bipolar type I}  Excluded if number of episodes missing or bipolar state missing | Single episode, recurrent depression and bipolar type I should include all depression cases with valid responses |  |
| Depression | ***Variant: Depression single episode triggered by loss*** | {depression single episode}  AND  worst depression start within two months of traumatic event (20447) = yes | Cases of single episode triggered by loss could be selectively excluded for some analyses, although likely to exclude some true cases of major depressive episode |  |
| Depression | **Score: PHQ-9.**  score items 0-4 and sum  (Little interest or pleasure in doing things 20514, Feeling down, depressed, or hopeless 20510, Trouble sleeping 20517, Feeling tired 20519, Poor appetite or overeating 20511, Feeling bad about yourself 20507, Trouble concentrating 20508, Moving or speaking slowly or fidgety or restless 20518, Thoughts that you would be better off dead 20513) | (“20514, 20510, 20517, 20519, 20511, 20507, 20508, 20518, 20513”) (subtract 9 if items scored 1-5)  If value missing, count as “0” when scoring 0-4 | *Kroenke K, Spitzer RL, Williams JB, Löwe B. The patient health questionnaire somatic, anxiety, and depressive symptom scales: a systematic review. Gen Hosp Psychiatry. 2010;32(4):345-59.* |  |
| Depression | **Case: Current depression.**  PHQ +ve and CIDI+ve  Reports symptoms in the last two weeks that have bothered them. Current depression is indicated by five or more items marked to bother at or above a certain intensity: “more than half of days” for first eight items, “some days” for last item. | {depression ever}  AND  Total symptoms endorsed as occurring more than half days (or some or more days for last item) ≥ 5   - Little interest or pleasure in doing things 20514, Feeling down, depressed, or hopeless 20510, Trouble sleeping 20517, Feeling tired 20519, Poor appetite or overeating 20511, Feeling bad about yourself 20507, Trouble concentrating 20508, Moving or speaking slowly or fidgety or restless 20518, Thoughts that you would be better off dead 20513 | For identifying likely depression, can use “diagnostic algorithm” based on DSM criteria, alternatively total score. This is using “diagnostic algorithm”  *Manea L, Gilbody S, McMillan D. Optimal cut-off score for diagnosing depression with the Patient Health Questionnaire (PHQ-9): a meta-analysis. CMAJ. 2012;184(3):E191-E6* |  |
| Depression | **Control: Current depression.**  PHQ score ≤5 | PHQ score ≤5 | A score of above 5 on PHQ can be used as a cut-off for mild depression. Therefore this control group excludes people with possible mild depression, as well as those who meet full criteria in the diagnostic algorithm.  *Manea L, Gilbody S, McMillan D. Optimal cut-off score for diagnosing depression with the Patient Health Questionnaire (PHQ-9): a meta-analysis. CMAJ. 2012;184(3):E191-E6* |  |
| Depression | ***Variant: Current severe depression***  *As current depression (above) with PHQ score > 15* | {depression current}  AND  PHQ score >15 | *Manea L, Gilbody S, McMillan D. Optimal cut-off score for diagnosing depression with the Patient Health Questionnaire (PHQ-9): a meta-analysis. CMAJ. 2012;184(3):E191-E6* |  |
| Mania  ** | **Symptoms: Hypomania / Mania.**  Endorses features of hypomania / mania lasting for a week or more, whether or not they were disruptive, and whether or not a depression ever case. Requires “High-hyper” plus three other symptoms or “Irritable” plus four other symptoms | High/Hyper 20501 = 01 OR Irritable 20502 = 01  AND  Four features from:   - High/Hyper 20501; Active 20548(01); Talkative 20548(02); Less sleep 20548(03); Creative/ideas 20548(04); Restless 20548(5); Confident 20548(6); Thoughts racing 20548(7); Easily distracted 20548(8)   AND  Duration 20492 = A week or more | Based on DSM-IV definition of hypo/mania. This includes likely cases of bipolar affective disorder type I, possible bipolar type II (where symptoms last a week), recurrent mania without clear depression, and antidepressant-induced symptoms of hypomania / mania.  *Smith DJ, Nicholl BI, Cullen B, Martin D, Ul-Haq Z, Evans J, et al. Prevalence and characteristics of probable major depression and bipolar disorder within UK biobank: cross-sectional study of 172,751 participants. PLoS One. 2013;8(11):e75362* |  |
| Mania | **Case: Bipolar affective disorder type I.**  Ever manic/hyper or irritable, plus at least three other features (four if never manic/hyper), plus duration a week or more, plus symptoms caused significant problems. Requires also to be case for depression ever. | Case {depression ever}  AND  High/Hyper 20501 = 01 OR Irritable 20502 = 01  AND  Four features from:   - High/Hyper 20501; Active 20548(01); Talkative 20548(02); Less sleep 20548(03); Creative/ideas 20548(04); Restless 20548(5); Confident 20548(6); Thoughts racing 20548(7); Easily distracted 20548(8)   AND  Duration 20492 = A week or more  AND  Symptoms caused problem 20493 = yes | Case for depression is not required in DSM-IV diagnostic criteria, but is added here to improve the positive predictive value of the test (see text and references). This definition does not exclude antidepressant-induced mania.  *Cerimele et al. The prevalence of bipolar disorder in primary care samples: a systematic review, General Hospital Psychiatry 36 (2014) 19-25*  *Carvalho, A. F., Y. Takwoingi, et al. (2015). "Screening for bipolar spectrum disorders: a comprehensive meta-analysis of accuracy studies." Journal of affective disorders* ***172****: 337-346* |  |
| Mania | ***Variant: Case bipolar type II***  *As above, without disruption from symptoms* | *Case {depression ever}*  *AND*  *High/Hyper 20501 = 01 OR Irritable 20502 = 01*  *AND*  *Four features as above*  *AND*  *Duration 20492 = A week or more* | There is less agreement over the definition of bipolar affective disorder type II. DSM-IV criteria require symptoms for four days or more. Here is one week, so could be predicted to miss some cases. |  |
| Mania | **Control: Hypomania / Mania**  Not included in hypomania / mania symptoms, nor categorised as bipolar on last UKB classification, nor self-reported bipolar | NOT {hypomania/mania}  AND  NOT {categorised bipolar on last UKB categorisation 20126 = 1 or 2}  AND  NOT {self-reported bipolar 20544=10} |  |  |
| Anxiety | | | | |
| GAD  ** | **Case: GAD Ever.**  Excessive worrying about a number of issues, occurring most days for six months and difficult to control, with three or more somatic symptoms and functional impairment. | Worried tense of anxious (20421) = Yes  AND  Duration (20420) >= 6 months or All my life  AND  Most days (20538) = Yes  AND  Excessive: More than most (20425) OR Stronger than most (20542)  AND  Number of issues: More than one thing (20543) OR Different worries (20540)  AND  Difficult to control: Difficult to stop worrying (20541) OR Couldn’t put it out of mind (20539) OR Difficult to control (20537)  AND  Functional impairment: Role interference (20418) = Some or A lot  AND  3 somatic symptoms out of:  Restless. 20426; Keyed up or on edge. 20423; Easily tired. 20429; Having difficulty keeping your mind on what you were doing. 20419; More irritable than usual. 20422; Having tense, sore, or aching muscles. 20417; Often having trouble falling or staying asleep. 20427 | CIDI-SF (Composite International Diagnostic Interview – Short Form), GAD module, lifetime version. Scored based on DSM definition of GAD  *Kessler RC, Andrews G, Mroczek D, Ustun B, Wittchen HU. The World Health Organization composite international diagnostic interview short‐form (CIDI‐SF). Int J Methods Psychiatr Res. 1998;7(4):171-85.*  *National Institute for Health and Clinical Excellence. Generalised anxiety disorder and panic disorder in adults: management. NICE Clinical Guideline CG113 (available at https://www.nice.org.uk/guidance/cg113) 2011* |  |
| GAD | **Control: GAD ever.**  Not meeting criteria for GAD ever nor scoring over low cut-off for GAD-7 | NOT case {GAD ever}  AND  GAD-7 score < 5 | Excluding those that screen positive for mild anxiety means that there is greater confidence that this group have not had anxiety disorder |  |
| GAD | **Score: GAD-7**  Score 0-3 and sum  a) Feeling nervous, anxious or on edge 20506  b) Not being able to stop or control worrying 20509  c) Worrying too much about different things 20520  d) Trouble relaxing 20515  e) Being so restless that it is hard to sit still 20516  f) Becoming easily annoyed or irritable 20505  g) Feeling afraid as if something awful might happen 20512 | Sum {Feeling nervous, anxious or on edge 20506, Not being able to stop or control worrying 20509, Worrying too much about different things 20520, Trouble relaxing 20515, Being so restless that it is hard to sit still 20516, Becoming easily annoyed or irritable 20505, Feeling afraid as if something awful might happen 20512} 0,1,2,3  (nb in biobank coded 1-4, subtract 7 to adjust)  If item missing, score 0 when scoring 0-3 | *Kroenke K, Spitzer RL, Williams JB, Löwe B. The patient health questionnaire somatic, anxiety, and depressive symptom scales: a systematic review. Gen Hosp Psychiatry. 2010;32(4):345-59* |  |
| GAD | **Case: Current anxiety.**  GAD-7 score ≥10 and case GAD ever | Case {GAD ever}  AND  GAD-7 score ≥10  Where each item scored 0-3 | Can be scored with cut-offs for mild, moderate and severe, with cut-offs at 5, 10 and 15. 10 chosen to represent moderate.  *Kroenke K, Spitzer RL, Williams JB, Löwe B. The patient health questionnaire somatic, anxiety, and depressive symptom scales: a systematic review. Gen Hosp Psychiatry. 2010;32(4):345-59* |  |
| PTSD | **Score: PCL-6**  Sum of scores on questions representing the core symptoms of PTSD  Score 1-5 and sum  [20497Repeated disturbing thoughts of stressful experience in past month](http://biobank.ctsu.ox.ac.uk/crystal/field.cgi?id=20497)  [20498Felt very upset when reminded of stressful experience in past month](http://biobank.ctsu.ox.ac.uk/crystal/field.cgi?id=20498)  [20495Avoided activities or situations because of previous stressful experience in past month](http://biobank.ctsu.ox.ac.uk/crystal/field.cgi?id=20495)  [20496Felt distant from other people in past month](http://biobank.ctsu.ox.ac.uk/crystal/field.cgi?id=20496)  [20494Felt irritable or had angry outbursts in past month](http://biobank.ctsu.ox.ac.uk/crystal/field.cgi?id=20494)  20508 Trouble concentrating (scored 1-4) | Sum {[20497Repeated disturbing thoughts of stressful experience in past month](http://biobank.ctsu.ox.ac.uk/crystal/field.cgi?id=20497), [20498Felt very upset when reminded of stressful experience in past month](http://biobank.ctsu.ox.ac.uk/crystal/field.cgi?id=20498), [20495Avoided activities or situations because of previous stressful experience in past month](http://biobank.ctsu.ox.ac.uk/crystal/field.cgi?id=20495), [20496Felt distant from other people in past month](http://biobank.ctsu.ox.ac.uk/crystal/field.cgi?id=20496), [20494Felt irritable or had angry outbursts in past month](http://biobank.ctsu.ox.ac.uk/crystal/field.cgi?id=20494)} 1,2,3,4,5 + {20508 Trouble concentrating} 1,2,3,4  (nb biobank coded 0-4, subtract 5 to adjust) | Using PHQ item for concentration, scores out of 29 (conventionally scores out of 30), and will make it slightly harder to reach conventional threshold.  *Lang AJ, Stein MB. An abbreviated PTSD checklist for use as a screening instrument in primary care. Behaviour research and therapy. 2005;43(5):585-94* |  |
| PTSD | **Case: PTSD.**  PCL-6 sum of scores 14 or greater is positive screen | ([20497Repeated disturbing thoughts](http://biobank.ctsu.ox.ac.uk/crystal/field.cgi?id=20497) + [20498Felt very upset when reminded](http://biobank.ctsu.ox.ac.uk/crystal/field.cgi?id=20498) + [20495Avoided activities or situations](http://biobank.ctsu.ox.ac.uk/crystal/field.cgi?id=20495) + [20496Felt distant](http://biobank.ctsu.ox.ac.uk/crystal/field.cgi?id=20496) + [20494Felt irritable or had angry outbursts](http://biobank.ctsu.ox.ac.uk/crystal/field.cgi?id=20494) + 20508 Trouble concentrating)>13 | Does not currently require catastrophic trauma, but refers to “stressful event” in the text of the questions as this is not an exhaustive list of possible trauma. |  |
| PTSD | **Control: PTSD.**  PCL-6 sum of scores 13 or less is positive screen. Include those who do not complete PCL-6 due to stop rule. | ([20497Repeated disturbing thoughts](http://biobank.ctsu.ox.ac.uk/crystal/field.cgi?id=20497) + [20498Felt very upset when reminded](http://biobank.ctsu.ox.ac.uk/crystal/field.cgi?id=20498) + [20495Avoided activities or situations](http://biobank.ctsu.ox.ac.uk/crystal/field.cgi?id=20495) + [20496Felt distant](http://biobank.ctsu.ox.ac.uk/crystal/field.cgi?id=20496) + [20494Felt irritable or had angry outbursts](http://biobank.ctsu.ox.ac.uk/crystal/field.cgi?id=20494) + 20508 Trouble concentrating)>13 |  |  |
| Other symptoms | | | | |
| Psychotic experiences ** | **Symptom: Psychotic experience.**  Endorsed possible hallucination or delusion | Heard unreal voice 20463 = yes  OR  Saw unreal vision 20471 = yes  OR  Believed unreal conspiracy 20468 = yes  OR  Believed unreal communication or signs 20474 = yes | Adapted by group from CIDI questions  *Nuevo R, Chatterji S, Verdes E, Naidoo N, Arango C, Ayuso-Mateos JL. The Continuum of Psychotic Symptoms in the General Population: A Cross-national Study. Schizophrenia Bulletin. 2012;38(3):475-85* |  |
| Psychotic experiences | **Symptom: Recent psychotic experience.**  Reports hallucination or delusion in the last year | Frequency in last year 20467>0 |  |  |
| Psychotic experiences | **Control: Psychotic experience.**  Not endorsing psychotic illness or reporting symptoms | NOT Endorsed diagnosis 20544 of schizophrenia [2] or other psychotic illness [3]  AND  NOT {ever hallucination} OR {ever delusion} |  |  |
| Self-harm | **Case: Life not worth living.**  Ever felt life not worth living | 20479 life NWL = yes (1 or 2) |  |  |
| Self-harm | **Case: Self harm.**  Ever harmed self, whether or not meant to die | 20480 Self harmed = Yes |  |  |
| Alcohol and addiction | | | | |
| Alcohol | **Score: AUDIT**  Asks about “in the last year” apart from last two questions.  (Note coding on UKB is from 1-5, so requires adjustment)  Sum individual scores | PART 1 Hazard: Frequency (scored 0-4) 20414, typical drinks (score 0-4) 20403, six or more drinks (scored 0-4) 20416  PART 2 Dependence: Unable to stop (scored 0-4) 20413, failed to do what expected due to drinking (scored 0-4) 20407, needed to drink first thing (scored 0-4) 20412  PART 3 Harm: Guilt due to drinking (scored 0-4) 20409, unable to remember due to drink (scored 0-4) 20408, injury due to drinking ever (scored 0,2,4) 20411, advice to cut down ever (scored 0,2,4) 20405 | Can be scored using algorithm or cut-offs, with more literature on the latter approach. Using cut-off of 8 is to indicate likelihood of moderate severity, 16 indicates severe, and lower cut-offs have been used to identify hazardous drinking (as opposed to drinking already causing harm).  *Reinert, D. F. and J. P. Allen (2007). "The alcohol use disorders identification test: an update of research findings." Alcoholism: Clinical and Experimental Research 31(2): 185-199* |  |
| Alcohol | **Case: Alcohol Use Disorder.**  Alcohol use disorder of moderate severity is predicted by score of 8 or more. | {AUDIT score} ≥8 | *Babor, T. F., J. C. Higgins-Biddle, et al. (2001). "AUDIT: The alcohol use disorders identification test: Guidelines for use in primary health care."* |  |
| Alcohol | **Control: Alcohol Use Disorder.**  Uses inverse of the algorithmic diagnosis of hazardous drinking from AUDIT, excluding those who reported alcohol addiction in this questionnaire or reported at baseline they had stopped drinking due to illness, on drs advice or as a health precaution | AUDIT –ve ((Drinks alcohol 30414 = 0) OR **(**Typical drinks 20403 = “1 or 2” AND Six or more 20416 = “Never”))  AND  NOT {ever alcohol dependence}  AND  NOT reason for reducing amount of alcohol drunk 2664 = “ill health”, “doctor’s advice”or “health precaution” [1,2or3] | This is particularly strict control group to avoid including participants recovering from alcohol harm/dependence in the definition. |  |
| Addiction | **Case: Addiction ever.**  Endorses “Ever addicted to any substance or behaviour” | “Ever addicted to any substance or behaviour” 20401=1 |  |  |
| Addiction | **Case: Substance addiction.**  Endorses ever addicted to alcohol or drugs or medication. | Alcohol 20406 = Yes (1)  OR  Illicit/recreational drugs = Yes (1)  OR  Medication = Yes (1) |  |  |
| Addiction | **Case: Current addiction:**  Endorses “addiction or dependence ongoing” | 20457=1 or 20504=1 or 20415=1 or 20432=1 |  |  |
| Addiction | **Case Alcohol dependence ever.**  Endorses “physically dependent on alcohol” | 20404=1 |  |  |
| Addiction | **Control: Addiction ever.**  Not endorsing addiction, or other indicators of misuse: screening AUDIT in severe alcohol use disorder range or daily use of cannabis | NOT {ever addiction}  AND  NOT {AUDIT score >16}  AND  {daily cannabis} defined below |  |  |

## Appendix 3 ICD-10 codes used for hospital data-linkage

Depression

"F320", "F321", "F322", "F323", "F328", "F329", "F330", "F331", "F332", "F333", "F334", "F338", "F339"

Anxiety

"F400", "F401", "F402", "F408", "F409", "F410", "F411", "F412", "F413", "F418", "F419", "F420", "F421", "F422", "F428", "F429", "F430", "F431", "F432", "F438", "F439", "F440", "F441", "F442", "F443", "F444", "F445", "F446", "F447", "F448", "F449", "F450", "F451", "F452", "F453", "F454", "F458", "F459", "F480", "F481", "F488", "F489"

Bipolar affective disorder

"F300", "F301", "F302", "F308", "F309", "F310", "F311", "F312", "F313", "F314", "F315", "F316", "F317", "F318", "F319"

Psychosis or psychotic symptoms

"F20", "F200", "F201", "F202", "F203", "F204", "F205", "F206", "F208", "F209", "F21", "F220", "F228", "F229", "F23", "F230", "F231", "F232", "F233", "F238", "F239", "F24", "F250", "F251", "F252", "F258", "F259", "F28", "F29", "F302", "F312", "F315", "F323", "F333"

## Appendix 4 UKB Medication codes used

Antidepressants

(attr. Christopher Hübel and Héléna A. Gaspar)

| Code in UKB | Drug name |
| --- | --- |
| 1140879616 | amitriptyline |
| 1140921600 | citalopram |
| 1140879540 | fluoxetine |
| 1140867878 | sertraline |
| 1140916282 | venlafaxine |
| 1140909806 | dosulepin |
| 1140867888 | paroxetine |
| 1141152732 | mirtazapine |
| 1141180212 | escitalopram |
| 1140879634 | trazodone |
| 1140867876 | prozac |
| 1140882236 | seroxat |
| 1141190158 | cipralex |
| 1141200564 | duloxetine |
| 1140867726 | lofepramine |
| 1140879620 | clomipramine |
| 1140867818 | nortriptyline |
| 1140879630 | imipramine |
| 1140879628 | dothiepin |
| 1141151946 | cipramil |
| 1140867948 | amitriptyline |
| 1140867624 | prothiaden |
| 1140867756 | trimipramine |
| 1140867884 | lustral |
| 1141151978 | reboxetine |
| 1141152736 | zispin |
| 1141201834 | cymbalta |
| 1140867690 | anafranil |
| 1140867640 | doxepin |
| 1140867920 | moclobemide |
| 1140867850 | phenelzine |
| 1140879544 | fluvoxamine |
| 1141200570 | yentreve |
| 1140867934 | triptafen |
| 1140867758 | surmontil |
| 1140867914 | tranylcypromine |
| 1140867820 | allegron |
| 1141151982 | edronax |
| 1140882244 | molipaxin |
| 1140879556 | mianserin |
| 1140867852 | nardil |
| 1140867860 | faverin |
| 1140917460 | nefazodone |
| 1140867938 | amitriptyline+chlordiazepoxide |
| 1140867856 | isocarboxazid |
| 1140867922 | manerix |
| 1140910820 | maoi |
| 1140882312 | sinequan |
| 1140867944 | tranylcypromine+trifluoperazine |
| 1140867784 | ludiomil |
| 1140867812 | norval |
| 1140867668 | tryptizol |

Antipsychotics

(attr. Christopher Hübel and Héléna A. Gaspar)

| 1140928916 | olanzapine |
| --- | --- |
| 1141152848 | quetiapine |
| 1140867444 | risperidone |
| 1140879658 | chlorpromazine |
| 1140868120 | trifluoperazine |
| 1141153490 | amisulpride |
| 1140867304 | sulpiride |
| 1141152860 | seroquel |
| 1140867168 | haloperidol |
| 1141195974 | aripiprazole |
| 1140867244 | stelazine |
| 1140867152 | depixol |
| 1140909800 | flupentixol |
| 1140867420 | clozapine |
| 1140879746 | promazine |
| 1141177762 | risperdal |
| 1140867456 | modecate |
| 1140867952 | fluanxol |
| 1140867150 | flupenthixol |
| 1141167976 | zyprexa |
| 1140882100 | zuclopenthixol |
| 1140867342 | clopixol |
| 1140863416 | largactil |
| 1141202024 | abilify |
| 1140882098 | fluphenazine |
| 1140867184 | haldol |
| 1140867092 | serenace |
| 1140882320 | clozaril |
| 1140910358 | cpz |
| 1140867208 | perphenazine |
| 1140909802 | levomepromazine |
| 1140867134 | pericyazine |
| 1140867306 | dolmatil |
| 1140867210 | fentazin |
| 1140867398 | fluphenazine |
| 1140867078 | benperidol |
| 1140867218 | pimozide |
| 1141201792 | zaponex |
| 1141200458 | denzapine |
| 1140867136 | neulactil |
| 1140879750 | thioridazine |
| 1140867180 | dozic |
| 1140867546 | fluspirilene |
| 1140928260 | panadeine |
| 1140927956 | sertindole |

Lithium

| 1140867490 | lithium product |
| --- | --- |
| 1140867494 | camcolit 250 tablet |
| 1140867498 | liskonum 450mg m/r tablet |
| 1140867500 | phasal 300mg m/r tablet |
| 1140867504 | priadel 200mg m/r tablet |
| 1140867518 | litarex 564mg m/r tablet |
| 1140867520 | li-liquid 5.4mmol/5ml oral solution |
